# Stratification to Neoadjuvant Radiotherapy in Rectal Cancer by Regimen and Transcriptional Signatures

**DOI:** 10.1158/2767-9764.CRC-23-0502

**Published:** 2024-07-18

**Authors:** Umair Mahmood, Andrew Blake, Sanjay Rathee, Leslie Samuel, Graeme Murray, David Sebag-Montefiore, Simon Gollins, Nicholas P. West, Rubina Begum, Simon P. Bach, Susan D. Richman, Phil Quirke, Keara L. Redmond, Manuel Salto-Tellez, Viktor H. Koelzer, Simon J. Leedham, Ian Tomlinson, Philip D. Dunne, Francesca M. Buffa, S:CORT consortium, Tim S. Maughan, Enric Domingo

**Affiliations:** 1 Department of Oncology, Medical Science Division, University of Oxford, Oxford, United Kingdom.; 2 School of Medicine, Medical Sciences and Nutrition, University of Aberdeen, Aberdeen, United Kingdom.; 3 Leeds Institute of Medical Research, University of Leeds, Leeds, United Kingdom.; 4 North Wales Cancer Treatment Centre, Besti Cadwaladr University Health Board, Bodelwyddan, United Kingdom.; 5 Lingen Davies Cancer Centre, Shrewsbury and Telford Hospital NHS Trust, Shrewsbury, United Kingdom.; 6 Cancer Research & University College London Clinical Trial Unit, London, United Kingdom.; 7 Cancer Research UK Clinical Trials Unit, University of Birmingham, Birmingham, United Kingdom.; 8 The Patrick G Johnston Centre for Cancer Research, Queens University Belfast, Belfast, United Kingdom.; 9 Department of Pathology and Molecular Pathology, University Hospital Zurich, University of Zurich, Zurich, Switzerland.; 10 Department of Oncology and Nuffield Department of Medicine, University of Oxford, Oxford, United Kingdom.; 11 Wellcome Trust Centre for Human genetics, Nuffield Department of Medicine, University of Oxford, Oxford, United Kingdom.; 12 Department of Computing Sciences, Bocconi University, and Bocconi Institute for Data Science and Analytics (BIDSA), Milano, Italy.; 13 Department of Molecular and Clinical Cancer Medicine, University of Liverpool, Liverpool, United Kingdom.; 14 Cancer Research UK Scotland Centre, Edinburgh, United Kingdom.

## Abstract

Response to neoadjuvant radiotherapy (RT) in rectal cancer has been associated with immune and stromal features that are captured by transcriptional signatures. However, how such associations perform across different chemoradiotherapy regimens and within individual consensus molecular subtypes (CMS) and how they affect survival remain unclear. In this study, gene expression and clinical data of pretreatment biopsies from nine cohorts of primary rectal tumors were combined (*N* = 826). Exploratory analyses were done with transcriptomic signatures for the endpoint of pathologic complete response (pCR), considering treatment regimen or CMS subtype. Relevant findings were tested for overall survival and recurrence-free survival. Immune and stromal signatures were strongly associated with pCR and lack of pCR, respectively, in RT and capecitabine (Cap)/5-fluorouracil (5FU)–treated patients (*N* = 387), in which the radiosensitivity signature (RSS) showed the strongest association. Upon addition of oxaliplatin (Ox; *N* = 123), stromal signatures switched direction and showed higher chances to achieve pCR than without Ox (p for interaction 0.02). Among Cap/5FU patients, most signatures performed similarly across CMS subtypes, except cytotoxic lymphocytes that were associated with pCR in CMS1 and CMS4 cases compared with other CMS subtypes (p for interaction 0.04). The only variables associated with survival were pCR and RSS. Although the frequency of pCR across different chemoradiation regimens is relatively similar, our data suggest that response rates may differ depending on the biological landscape of rectal cancer. Response to neoadjuvant RT in stroma-rich tumors may potentially be improved by the addition of Ox. RSS in preoperative biopsies provides predictive information for response specifically to neoadjuvant RT with 5FU.

**Significance:**

Rectal cancers with stromal features may respond better to RT and 5FU/Cap with the addition of Ox. Within patients not treated with Ox, high levels of cytotoxic lymphocytes associate with response only in immune and stromal tumors. Our analyses provide biological insights about the outcome by different radiotherapy regimens in rectal cancer.

## Introduction

Colorectal cancer ranks the third most common tumor type in the Western world, with 30% of such cancers located in the rectum (1). Currently, the standard of care (SOC) for locally advanced rectal cancer is radiotherapy (RT) with or without different chemotherapy regimens followed by radical surgical resection. Tumor stages T3 to T4, nodal involvement, extramural venous invasion, and/or threatened circumferential margin are indications for neoadjuvant treatment (1, 2). However, response to combined chemoradiotherapy varies, and treatment is associated with both short-term toxicity (acute diarrhea, 12%; dermatologic defects, 11%) and long-term side effects (chronic diarrhea and small bowel obstruction, 9%; ref. 3). Pathologic complete response (pCR) occurs in only 10% to 20% of patients with rectal cancer following neoadjuvant therapy (1), with 30% to 40% of patients showing no or minimal evidence of tumor regression (4). Hence, there is a significant unmet medical need to prescribe SOC treatments tailored to the biological background of a given tumor. Currently, decisions for neoadjuvant treatment in rectal cancer are mostly guided by MRI which is less accurate in distinguishing metastatic from nonmetastatic lymph nodes than pathologic examination (1, 5). Clinical staging and assessment of the preoperative tumor biopsy can guide decisions about the requirement of the treatment, but it has limited predictive utility for response to treatment, from long/short pelvic radiation alone or combined with fluoropyrimidines with or without oxaliplatin (Ox) or other chemotherapies. Currently, a lack of clinically validated predictive biomarkers for neoadjuvant chemoradiation in rectal cancer needs to be addressed urgently to improve outcome and life quality (1, 6).

A transcriptomics-driven bioinformatic approach can be used to identify molecular features that can guide the selection of patients who may obtain a clinical benefit if matched with the appropriate SOC treatment (7). Multiple studies have used such approaches to identify molecular features to classify patients with colorectal cancer to aid clinical decisions such as consensus molecular subtypes (CMS; ref. 8) and the colorectal cancer intrinsic subtype (CRIS) classifiers (7). Some transcriptomic signatures have been reported as stratifiers for RT in rectal cancer but mostly without validation (9), and none of them have been developed further. However, using large multiomic datasets and sophisticated methodology, we have recently reported a new transcriptomic radiosensitive signature (RSS) that can predict pCR with high accuracy upon specific treatment of RT with fluoropyrimidines (9). Notably, there was a strong interaction of RSS with microenvironment features that are associated with higher likelihood of response: high levels of cytotoxic lymphocytes, the immune subtype CMS1, and low fibroblast TGFβ response signature (F-TBRS). This is fully consistent with the independent association of imCMS1 and imCMS4, which are derived from histopathology images (10), with pCR and lack of pCR in the same cohorts (11). These findings highlight that complete responding tumors are biologically distinct from noncomplete responders, which may aid patient stratification to clinically appropriate treatments.

Following our recent results which were limited to patients receiving the combination of long-course RT (45–50.4 Gy in 25 fractions) with single-agent fluoropyrimide [capecitabine (Cap) or 5-fluorouracil (5FU)], here we have combined transcriptomic data of pretreatment rectal biopsies from different private and public datasets, aiming to explore additional transcriptomic signatures, assess them by specific RT-related regimens, evaluate performance within CMS subtypes, and investigate their survival effects. To the best of our knowledge, this study assembling 826 cases represents by far the largest transcriptomic dataset ever compiled in this clinical setting.

## Materials and Methods

### Cohort and patient selection

Datasets from three clinical trials (ARISTOTLE, COPERNICUS, and TREC) and one community-based cohort (Grampian) were available through the Stratification in Colorectal Cancer (S:CORT) consortium. In addition, a review of publicly available transcriptomic microarray data in the Gene Expression Omnibus (GEO) was performed. The inclusion criterion was that datasets had to contain pretreatment biopsies of rectal cancer tissue with known pCR status and whole-transcriptome data. Seven public repositories were identified, but two were discarded: GSE53781 (not whole-genome transcriptome) and GSE68204 (partial missing expression data). Normal and posttreatment resection cases were excluded. A total of nine cohorts were finally included in the analysis with 826 useful cases (Supplementary Table S1). Clinical details for each cohort are available as Supplementary Methods.

Data for pretreatment T and N stages, pCR, and overall survival/recurrence-free survival (OS/RFS) were obtained from S:CORT or from metadata available through GEO. Additional clinical data from GSE94104 were provided by the original authors (12).

All S:CORT patients provided written informed consent for further research to be undertaken on samples. S:CORT cohorts were approved by the National Research Service in the United Kingdom (ref. 15/EE/0241).

### Grampian survival data

Grampian patients were followed up locally by checking six monthly clinical reviews, blood tests and imaging from liver ultrasound scans, and annual whole-body CT scanning. Recurrences were detected via symptoms, blood tests, or imaging. If a CT scan did not reveal recurrence but showed abnormal blood tests, a further fluorodeoxyglucose-PET scan was performed.

### Pretreatment sample processing

All subjects underwent pretreatment rectal biopsy procedures in which tissue was either fresh frozen or formalin-fixed, paraffin-embedded (Supplementary Table S1). Hematoxylin and eosin staining was performed on these specimens for a pathologist to review and to mark areas of sufficient tumor quality and quantity for molecular profiling.

### RNA profiling

Details for the transcriptome profiling of S:CORT samples have been reported (10). Briefly, tumor regions were marked on hematoxylin and eosin slides, which guided needle dissection on up to nine consecutive tissue sections at the Leeds Institute of Medical Research. These were then shipped to Queen’s University Belfast, where the tissues were digested, RNA extracted using High Pure RNA Paraffin kit (Roche), and hybridized on Almac XCel Arrays (Affymetrix). Arrays were scanned and stored as CEL files. Transcriptome data from publicly available cohorts were downloaded from GEO.

### Data aggregation

To compare the expression of samples across all available datasets in a standardized manner, all transcriptomes had to be combined into a single expression matrix. However, differences in microarray platforms, academic centers, and time periods can result in strong batch effects that need to be corrected. First, samples across all selected datasets that were not rectal pretreatment biopsies were excluded to minimize transcriptomic heterogeneity. Second, to maximize standardization within the four S:CORT cohorts that had been profiled with the same platform in the same laboratory but at different timeframes, all their CEL files were processed together with the gold standard robust multiarray average technique (13) using the R package “affy.” Third, genes not interrogated by the four different platforms were excluded to obtain 15,985 Entrez gene IDs in common across all samples. Gene-level data for each cohort were generated with the mean across all probesets linked to the same Entrez ID in the latest annotation file of each platform. Fourth, the gold standard combining batches technique (14) from the R package “sva” was used to correct technical batch effects by cohort while mitigating the loss of biological signals. Batch correction was performed only by cohort type and not via addition of other variables such as pCR.

### CMS and CRIS classification

CMSs were profiled by using two methods which were then compared. The first one was CMScaller (15) which works on both clinical and preclinical models so it may overcome CMS4 undercalling due to potential lack of microenvironment in biopsies (15). The second one was based on CMSclassifier (8) and combined random forest and single-sample predictor calls without applying any cutoff to the posterior probability to decrease the number of unclassified cases in formalin-fixed, paraffin-embedded cases (10, 16).

### Other RNA signatures

RNA-based signature profiles were derived from the combined transcriptome using the R packages originally provided or alternatively by replicating the same methods as in their original reports. These included the following published candidates (9):

• *Radiosensitivity index*: intrinsic radiosensitivity index derived from pan-cancer cell lines (17).
• *Hypoxia Buffa*: hypoxia metagene derived from solid tumors (18).
• *Microenvironment cell populations*-*counter* (*MCP*-*counter*)—*immune:* quantified abundance of eight immune signatures selected in our previous study: T cells, CD8 T cells, cytotoxic lymphocytes, NK cells, B lineage, monocytic lineage, myeloid dendritic cells, and neutrophils (19).
• *Estimation of STromal and Immune cells in MAlignant Tumors using Expression data*: estimated fraction of tumor purity (20).
• *F*-*TBRS*: TGFβ induced activation of fibroblasts (21).

The list was then expanded with additional signatures and also our recently published stratifiers:

• *MCP*-*counter—stromal:* quantified abundance of two types of stromal cells: fibroblasts and endothelial cells (22).
• *Endothelial* (*End*-*TBRS*), *macrophage*, *and T cell* (*T*-*TBRS*) *TGFβ response signatures*: TGFβ induced activation of endothelial cells, macrophages, and T cells (21).
• *Intestinal *S*tem *C*ell* (*ISC*) *score*: to evaluate adult ISCs (21).
• *Proliferation score*: to evaluate proliferation of crypt cells (23).
• *Late transient*-*amplifying score*: to evaluate late transient-amplifying (progenitor) cells (23).
• *RadioSensitivity Signature (RSS):* our recent stratifier for pCR trained in Grampian/ARISTOTLE and validated in GSE87211 in cases selected to be treated with RT and fluoropyrimidine (9). Biological scores were generated by subtracting the mean of genes positively associated with pCR to the mean of genes negatively associated with pCR.
• *Biological radiosensitivity classifier* (*BRSC*) *RNA*: biological prediction model developed by combining three RNA variables (F-TBRS, MCP cytotoxic lymphocytes, and CMS1; ref. 9).

### Statistical analyses

Logistic regression models were developed to evaluate the relationship between signatures and clinical variables with pCR. Signatures and clinical variables were scaled from 0 to 1 to make them comparable, whereas categorical variables were binarized. Multiple models were adjusted (e.g., by cohort, T stage, and N stage) or compared by interaction and hence detailed accordingly. FDR was employed using stringent Bonferroni correction when appropriate to correct for multiple testing (24). An AUC analysis was performed to assess the potential prediction value of response to treatment for the explored signatures.

The McNemar test was performed if there was a statistically significant difference between the proportion of unclassified samples of the single cohorts and the batch-corrected combined cohort. The χ^2^ test was performed to examine correlations between various clinical and molecular variables.

Kaplan–Meier estimators and Cox proportional hazards models were developed to assess RFS and OS. Only Grampian and GSE87211 cohorts had survival data available, and the study population selected were subjects treated with RT and Cap/5FU which formed a large pool of patients while minimizing clinical heterogeneity. The follow-up period was right censored at 60 months. Analysis was performed using both clinical and molecular signatures in univariate and multivariate models to minimize the impact of known confounders. The variable cohort in all these models showed high *P* values (*P* ≥ 0.435), suggesting minimal statistical effects. Statistical analyses were performed in R v4.0.3 using RStudio v1.4.1106.

### Data availability

The transcriptomic data from all four S:CORT cohorts used in this study are publicly available in the following links: https://www.s-cort.org/sites/default/files/exports/scort_ws3_grampian_export_84m9fndk/ws3_grampian_expression_raw.zip; https://www.s-cort.org/sites/default/files/exports/scort_ws3_aristotle_export_6ythgf78/ws3_aristotle_expression_raw.zip; https://www.s-cort.org/sites/default/files/exports/scort_ws3_trec_export_rudd8gjc/ws3_trec_expression_raw.zip; and https://www.s-cort.org/sites/default/files/exports/scort_ft2_export_cp4mbbe2/ft2_expression_raw.zip. The list of S:CORT sample IDs specifically used in this study is available at https://www.s-cort.org/sites/default/files/exports/sample_sheets/SCORT_samples_Mahmood_CRC.csv. Additional S:CORT data are available to all academic researchers on submission of a data request to the data access committee. For commercial agencies, the data will be made available through Cancer Research Horizons acting on behalf of the funders and consortium members. Scripts to reproduce results are available upon reasonable request.

## Results

### Data amalgamation

Transcriptomic data from nine individual cohorts were combined into a single dataset after correcting for batch effects (“Materials and Methods”). To check the performance of the resulting merged transcriptome, CMS calls based on CMScaller and CMSclassifer and CRIS calls were compared between each of the original single cohorts and the new combined dataset. In a paired comparison, most samples retained subtype classification following batch correction (Supplementary Fig. S1A–C). We selected CMS calls from CMScaller for all further analyses as it showed a lower number of unclassified cases than our CMSclassifier method (19.85% vs. 24.94%, respectively; Supplementary Fig. S1D). The number of CMS unclassified samples in single and combined cohorts was not different (22.76% vs. 19.85%, respectively, *P* = 0.22, McNemar test). However, for CRIS, it was statistically higher in single cohorts than in the combined dataset (10.17% vs. 6.53%, respectively, *P* = 0.01, McNemar test), suggesting that an increase in the number of samples may improve CRIS calling efficiency. Most unclassified samples from CMScaller were successfully classified as CRIS subtypes (Supplementary Fig. S1E) as previously published for rectal biopsies (12). These results provide evidence that biological signals were preserved after technical batch correction. Accordingly, the combined transcriptomic dataset was considered useful for data interrogation across cohorts.

### Characteristics of the study population

The clinical distribution of the 826 patients in the transcriptomic combined dataset indicated that several patients had pretreatment clinical T3 (*N* = 483, 58.47%) and N0 (*N* = 226, 27.36%) or N1 (*N* = 313, 37.89%) stage disease (Supplementary Table S2A). pCR of disease was achieved in 147 cases (17.80%; Supplementary Table S2B), in line with expectations for this clinical setting (1), although not stable across cohorts. Most patients received RT combined with Cap or FU (*N* = 401, 48.55%). A total of 137 patients received RT combined with Cap/FU and Ox (16.59%) or radiation only at different doses (*N* = 81, 9.81%). Remaining cases had rare or unknown RT-related regimen and were combined together and labeled as miscellaneous (*N* = 207, 25.06%). The distribution of CMS/CRIS subtypes, but not clinical variables, was similar across the four different treatment types (Table 1). However, such distribution was not always equal across all nine cohorts (Supplementary Table S2). Nevertheless, CMS and CRIS distributions were not statistically different (Supplementary Table S3). Although the distribution of T stage across CMS and CRIS subtypes was not statistically different (Supplementary Table S4), N stage was different in both of them (*P* = 0.003 and *P* < 0.001, respectively; Supplementary Table S5A and S5B), in which the poor prognostic subtypes CMS4 and CRIS-B show higher frequency of N2 stage cases. Overall, these results highlight substantial clinical heterogeneity of the whole dataset due to diverse selection on sample and treatment type in each individual set.

**Table 1:** Baseline characteristics of selected patients

Variable	Category	All cases	Cap+RT/5FU+RT	Cap+RT/5FU+RT with Ox	RT only	Miscellaneous chemoradiation
T stage	T1	8 (0.97%)	1 (0.25%)	0 (0.00%)	7 (8.64%)	0 (0.00%)
	T2	71 (8.60%)	33 (8.23%)	2 (1.46%)	35 (43.21%)	1 (0.48%)
	T3	483 (58.47%)	309 (77.06%)	109 (79.56%)	25 (30.86%)	40 (19.32%)
	T4	62 (7.51%)	48 (11.97%)	12 (8.76%)	1 (1.23%)	1 (0.48%)
	Missing	202 (24.46%)	10 (2.49%)	14 (10.22%)	13 (16.05%)	165 (79.71%)
N stage	N0	226 (27.36%)	133 (33.17%)	34 (24.82%)	57 (70.37%)	2 (0.97%)
	N1	313 (37.89%)	191 (47.63%)	77 (56.20%)	15 (18.52%)	30 (14.49%)
	N2	91 (11.02%)	67 (16.71%)	14 (10.22%)	0 (0.00%)	10 (4.83%)
	Missing	196 (23.73%)	10 (2.49%)	12 (8.76%)	9 (11.11%)	165 (79.71%)
pCR	Complete responders	147 (17.80%)	79 (19.7%)	11 (8.03%)	8 (9.88%)	49 (23.67%)
	Noncomplete responders	667 (80.75%)	322 (80.3%)	126 (91.97%)	68 (83.95%)	151 (72.95%)
	Missing	12 (1.45%)	0 (0.00%)	0 (0.00%)	5 (6.17%)	7 (3.38%)
Cohort	ARISTOTLE (control arm)	121 (14.65%)	121 (30.17%)	0 (0.00%)	0 (0.00%)	0 (0.00%)
	COPERNICUS	37 (4.48%)	0 (0.00%)	0 (0.00%)	0 (0.00%)	37 (17.87%)
	TREC	37 (4.48%)	0 (0.00%)	0 (0.00%)	37 (45.68%)	0 (0.00%)
	Grampian	223 (27.00%)	129 (32.17%)	50 (36.50%)	44 (54.32%)	0 (0.00%)
	GSE56699	57 (6.90%)	0 (0.00%)	0 (0.00%)	0 (0.00%)	57 (27.54%)
	GSE87211	203 (24.58%)	111 (27.68%)	87 (63.50%)	0 (0.00%)	5 (2.42%)
	GSE94104	40 (4.84%)	40 (9.98%)	0 (0.00%)	0 (0.00%)	0 (0.00%)
	GSE46862	69 (8.35%)	0 (0.00%)	0 (0.00%)	0 (0.00%)	69 (33.33%)
	GSE150082	39 (4.72%)	0 (0.00%)	0 (0.00%)	0 (0.00%)	39 (18.84%)
CMS^a^	CMS1	86 (10.41%)	40 (9.98%)	15 (10.95%)	13 (16.05%)	18 (8.70%)
	CMS2	152 (18.40%)	58 (14.46%)	29 (21.17%)	14 (17.28%)	51 (24.64%)
	CMS3	145 (17.55%)	80 (19.95%)	21 (15.33%)	14 (17.28%)	30 (14.49%)
	CMS4	279 (33.78%)	136 (33.92%)	55 (40.15%)	22 (27.16%)	66 (31.88%)
	Unclassified	164 (19.85%)	87 (21.70%)	17 (12.41%)	18 (22.22%)	42 (20.29%)
CRIS^b^	CRIS-A	226 (27.36%)	107 (26.68%)	31 (22.63%)	21 (25.93%)	67 (32.37%)
	CRIS-B	117 (14.16%)	52 (12.97%)	28 (20.44%)	11 (13.58%)	26 (12.56%)
	CRIS-C	158 (19.13%)	81 (20.20%)	15 (10.95%)	21 (25.93%)	41 (19.81%)
	CRIS-D	136 (16.46%)	67 (16.71%)	28 (20.44%)	13 (16.05%)	28 (13.53%)
	CRIS-E	135 (16.34%)	67 (16.71%)	24 (17.52%)	11 (13.58%)	33 (15.94%)
	Unclassified	54 (6.54%)	27 (6.73%)	11 (8.03%)	4 (4.94%)	12 (5.80%)
Total samples		826 (100.00%)	401 (100.00%)	137 (100.00%)	81. (100.00%)	207 (100.00%)

a Examination of the distribution of rectal cancer specimens by CMS subtype within the combined dataset based on treatment type yielded χ^2^ (9, *N* = 662) = 16.198, *P* = 0.06 (excluding missing and unclassified samples).

b Examination of the distribution of rectal cancer specimens by CRIS subtype within the combined dataset based on treatment type yielded χ^2^ (12, *N* = 772) = 17.808, *P* = 0.12 (excluding missing and unclassified samples).

### Signatures associated with pCR

**Figure 1:**
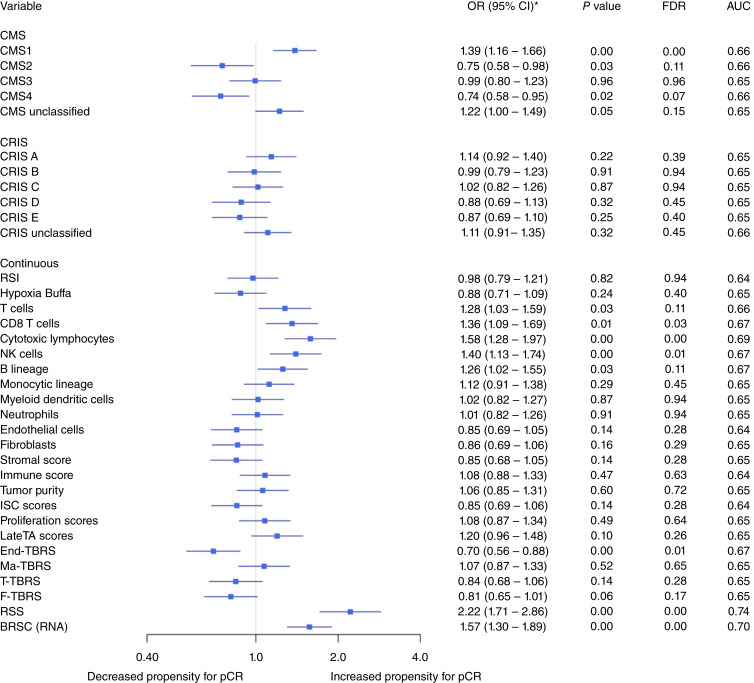
Multivariate logistic regression analysis of all subjects in the combined dataset adjusted by cohort, T stage, and N stage. *OR is reported as “OR per SD” to account for diverse distributions. LateTA score, late transient-amplifying score; Ma-TBRS, macrophage TGFβ response signature.

RNA signatures as listed in methods were interrogated for their association with the endpoint of pCR in the whole dataset (*N* = 616 patients; Fig. 1). CMS1 was significantly associated with pCR using the stringent FDR criteria [OR = 1.39 (95% confidence interval (CI), 1.16–1.66); *FDR* < 0.01]. Generally, immune signatures correlated with radiosensitivity, whereas cases with enrichment for stromal signatures were characterized by increased levels of radioresistance. Surprisingly, no signal was shown for hypoxia (18). From the additional signatures, our recent RSS signature was the best predictor [OR = 2.22 (95% CI, 1.71–2.86; *P* < 0.01)], followed by our biological BRSC [OR = 1.57 (95% CI, 1.30–1.89; *P* < 0.01)].

For our recent analysis reporting RSS, we specifically focused on patients undergoing a single chemoradiotherapy protocol (RT 5–50.4 Gy, Cap/5FU). Here, we expand this analysis in our large and diverse dataset selecting cases based on their different, specific RT-based regimens. Patients treated with RT+Cap/5FU (*N* = 387) demonstrated similar patterns of association with immune and stromal signatures showing significant associations with radiosensitivity and radioresistance, respectively (Supplementary Fig. S2A). However, although the previous analysis using all unselected cases had identified seven significant variables at the FDR level, this analysis on selected cases showed nine associations despite having lower sample size and hence lower statistical power. As expected, the best performers were RSS [OR = 2.85 (95% CI, 2.04–3.99; *P* < 0.01)] and BRSC [OR = 1.79 (95% CI, 1.42–2.27; *P* < 0.01)]. The other variables associated with radiosensitivity were CMS1, CD8 T cells, and cytotoxic lymphocytes, whereas those associated with radioresistance were CMS4, endothelial cells, ISC score, End-TBRS, T-TBRS, and F-TBRS (Supplementary Fig. S2A). As RSS and BRSC were trained on Grampian and ARISTOTLE, the same analysis was run excluding these two cohorts, which showed that they had the highest and second highest ORs, respectively, albeit not significant in this smaller subset (Supplementary Fig. S2B).

**Figure 2:**
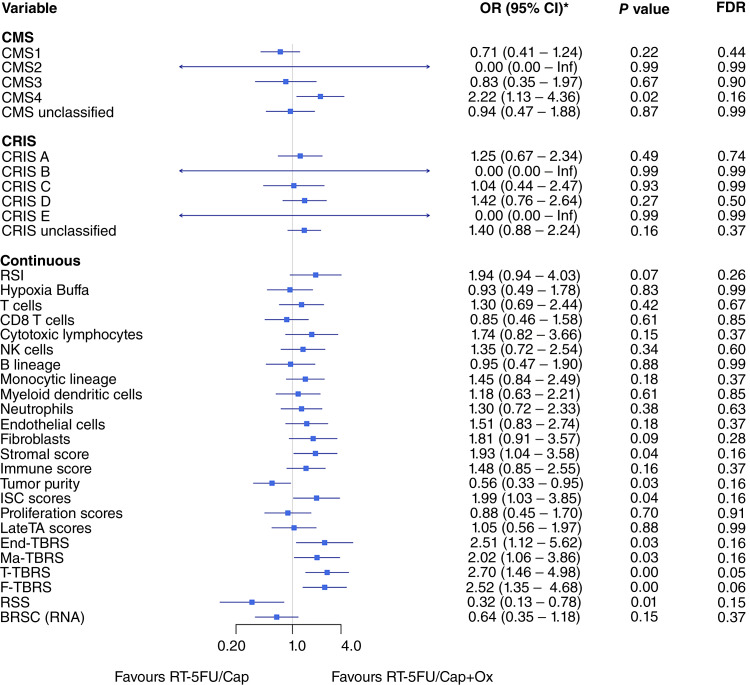
Multivariate logistic regression analysis of factors associated with complete response (CR) based on interaction with treatment type demonstrated a trend that favored RT+5FU/Cap+Ox recipients over RT+5FU/Cap patients with respect to CMS4 and overexpression of stromal signatures. However, RT+5FU/Cap+Ox recipients were less likely to achieve CR with increasing tumor purity and RSS scores. *OR is reported as “OR per SD” to account for diverse distributions. LateTA score, late transient-amplifying score; Ma-TBRS, macrophage TGFβ response signature.

We then analyzed 123 cases treated by RT, Cap/5FU, and Ox (Supplementary Fig. S3). No variable reached significant results for FDR in this modestly sized subset. However, the direction of the signals from both immune and stromal signatures was toward radiosensitivity. For example, four variables were significant by *P* value including the immune signature cytotoxic lymphocytes and the stromal signatures macrophage-TBRS, T-TBRS, and F-TBRS. Given the change in directionality in stromal signatures by the addition of Ox, we measured the interaction between both treatments (Fig. 2). The T-TBRS signature had significant results by FDR (FDR = 0.05), suggesting that high levels of activated stroma may result in higher chances of achieving pCR by the addition of Ox to fluoropyrimidine regimens.

Finally, we analyzed the smaller subset of 64 cases treated uniquely with RT. No statistically significant patterns of association with pCR were found (Supplementary Fig. S4).

### Associations with pCR within CMS subtypes

**Figure 3:**
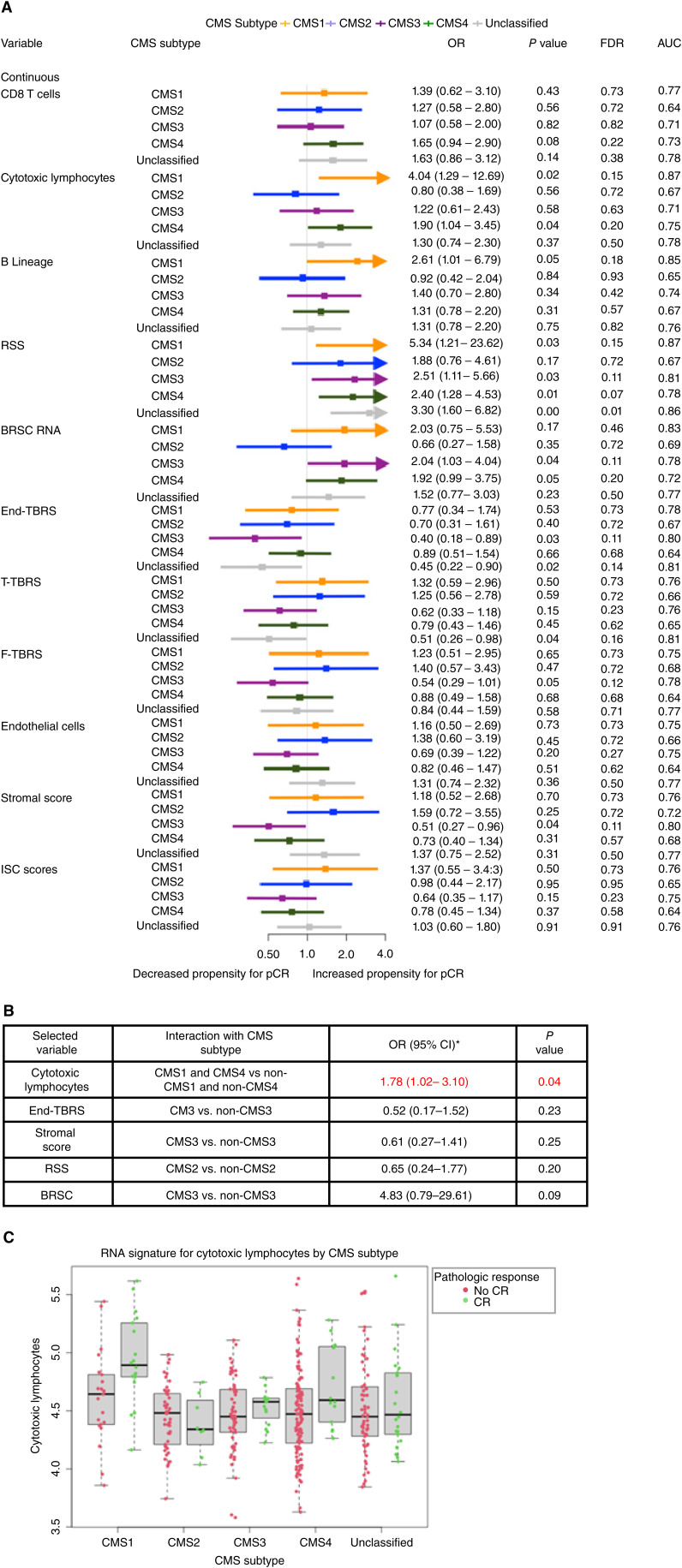
Subtype-specific multivariate analysis of factors associated with Cap+RT or 5FU+RT subjects demonstrated strong associations of immune, radiosensitivity, and stromal signatures with complete responders in specific CMS subtypes (**A**). Interaction-specific univariate regression analysis of signatures associated with complete response (CR) in specific CMS subtypes demonstrated cytotoxic lymphocytes as an important predictor of CR in this treatment cohort (**B** and **C**).

Our data provide strong evidence that microenvironment analysis is key to understand correlations with RT response. In order to better understand this association in the clinical cohorts under study, we aimed at interrogating our large dataset according to the immune/stromal status. For this purpose, we used CMS classification given the strong signals in our data and high relevance in colorectal cancer biology, in which CMS1 is the immune subtype and CMS4 is predominantly the stromal subtype with some level of immune activation. Given the heterogeneity found by the treatment regimen, only cases from the largest subset of patients treated by RT+Cap/5FU were used. Accordingly, these were further analyzed within each different CMS subtype. Eleven variables showed a *P* value below 0.05 (Fig. 3A). Patients classified as CMS1 and CMS4 subtypes with a high cytotoxic lymphocyte signature were more likely to achieve pCR [OR = 4.04 (95% CI, 1.29–12.69; *P* = 0.02) and OR = 1.90 (95% CI, 1.04–3.45; *P* = 0.04), respectively]. With regard to radiosensitivity-specific signatures, the elevated RSS score was strongly associated with pCR in all CMS subtypes with the exception of CMS2. However, higher BRSC scores depicted the strongest association in CMS3 samples [OR = 2.04 (95% CI, 1.03–4.04; *P* = 0.04)]. The stromal signature End-TBRS was linked with a reduced propensity for pCR among CMS3 [OR = 0.40 (95% CI, 0.18–0.89; *P* = 0.03)] or unclassified subtyped patients [OR = 0.45 (95% CI, 0.22–0.90; *P* = 0.02)]. Unclassified CMS cases with high T-TBRS were also associated with lack of pCR [OR = 0.51 (95% CI, 0.26–0.98; *P* = 0.04)]. Patients overexpressing stromal scores presented with a lower chance of pCR if they were classified as the CMS3 subtype [OR = 0.51 (95% CI, 0.27–0.96; *P* = 0.04)]. All remaining models were not significant (Fig. 3A).

Even though some signatures were linked with pCR within specific CMS subtypes, such analysis does not show whether this may be different compared with the other CMS subtypes. Accordingly, in order to identify subtype-specific trends, we performed interaction analyses based on different CMS combinations in variables with at least one CMS subtype showing a *P* value below 0.05 (Fig. 3B). High levels of cytotoxic lymphocytes were associated with a higher likelihood of pCR in CMS1 and CMS4 patients than other CMS subtypes [OR = 1.78 (95% CI, 1.02–3.10; *P* = 0.04); Fig. 3B and C]. No other combination of signature and CMS subtype was found.

A similar analysis was performed for the RT+5FU/Cap + Ox dataset, in which four transcriptomic signatures with a *P* value below 0.05 were selected for further subtype-specific analysis. However, none of the selected signatures demonstrated any significant association with pCR in this modestly sized treatment cohort (Supplementary Fig. S5).

### Survival in the RT+Cap/5FU patient population

**Figure 4:**
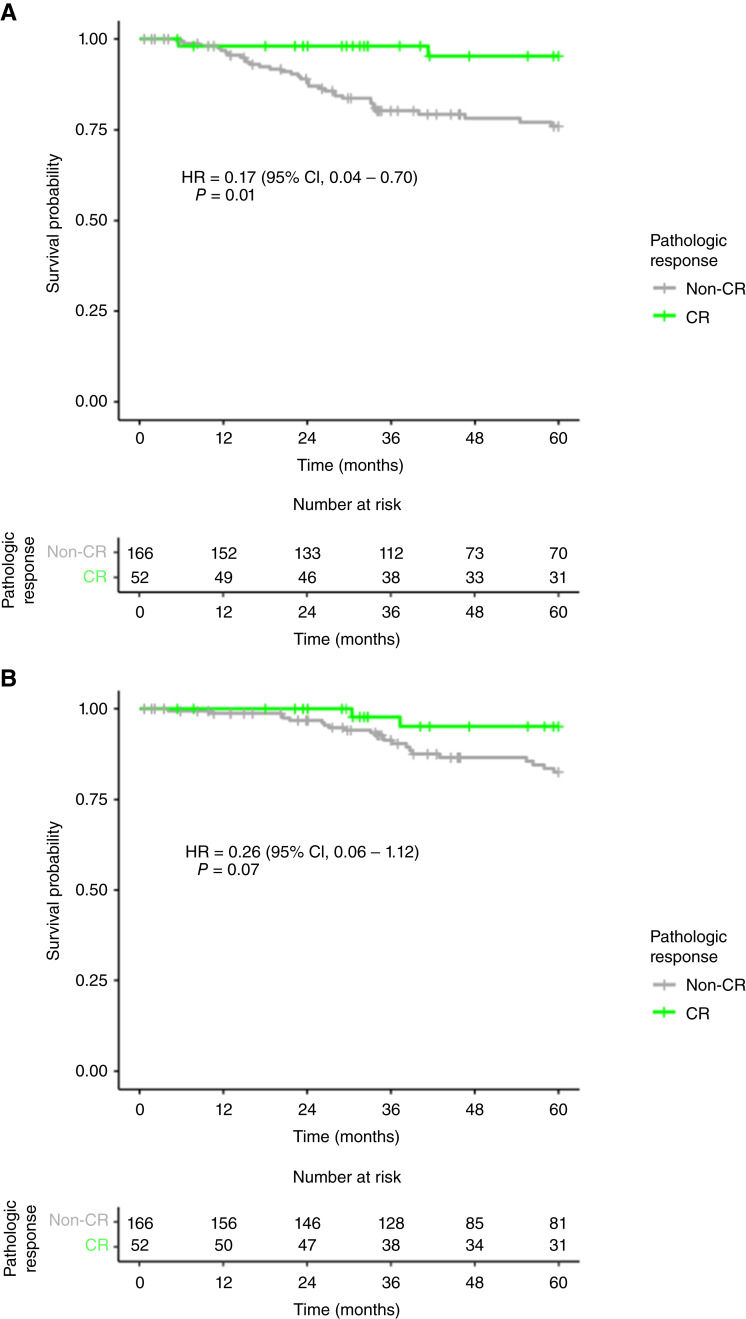
Stratification of survival outcomes by pCR by RFS (**A**) and OS (**B**).

Following our analyses for response to treatment, we aimed at exploring how our clinical and molecular variables may affect survival. We used the RT+Cap/5FU subset of patients from Grampian and GSE87211 with RFS and OS data available (*N* = 218). Better RFS was found by pCR in multivariate analysis [OR = 0.09 (95% CI, 0.01–0.69; *P* = 0.02); Fig. 4]. Baseline clinical variables were then checked (Table 2). Only N stage provided significantly worse OS in univariate analysis [OR = 1.95 (95% CI, 1.02–3.75; *P* = 0.04)] but not in an adjusted model. The variable cohort was also significant in univariate analysis [OR = 0.37 (95% CI, 0.14–0.98; *P* = 0.05)] but not after adjustment, suggesting some levels of heterogeneity in the survival data.

**Table 2:** RFS and OS outcomes in combined Grampian and GSE87211 cohorts (Cap+RT/5FU+RT subjects)

	RFS				OS			
	Univariate HR (95% CI)	*P* value	Adjusted HR (95% CI)*	*P* value	Univariate HR (95% CI)	*P* value	Adjusted HR (95% CI)*	*P* value
T stage	1.68 (0.73–3.87)	0.22	1.42 (0.59–3.42)	0.43	2.08 (0.75–5.81)	0.16	1.69 (0.62–4.62)	0.31
N stage	1.19 (0.69–2.05)	0.54	1.20 (0.66–2.17)	0.55	1.95 (1.02–3.75)	0.04	1.80 (0.95–3.41)	0.07
Cohort type (GSE87211 vs. Grampian)	1.75 (0.91–3.37)	0.09	1.83 (0.92–3.63)	0.09	0.37 (0.14–0.98)	0.05	0.38 (0.14–1.04)	0.06
CD8 T cells	1.17 (0.64–2.17)	0.61	1.18 (0.62–2.25)	0.61	1.24 (0.60–2.58)	0.56	1.34 (0.64–2.79)	0.44
Cytotoxic lymphocytes	0.83 (0.33–2.06)	0.69	0.85 (0.32–2.25)	0.75	1.14 (0.39–3.33)	0.81	1.74 (0.52–5.84)	0.37
ISC scores	2.24 (0.37–13.54)	0.38	1.52 (0.26–8.72)	0.64	1.17 (0.13–10.65)	0.89	0.80 (0.06–10.27)	0.87
End-TBRS	2.29 (0.69–7.64)	0.18	1.49 (0.38–5.87)	0.57	1.97 (0.43–9.00)	0.38	0.98 (0.23–4.24)	0.98
T-TBRS	4.09 (1.11–15.02)	0.04	2.70 (0.69–10.61)	0.15	3.05 (0.62–15.05)	0.17	1.24 (0.21–7.28)	0.82
F-TBRS	2.18 (0.82–5.83)	0.12	1.82 (0.67–4.93)	0.24	1.79 (0.55–5.80)	0.34	1.12 (0.30–4.20)	0.87
RSS	0.35 (0.17–0.70)	0.003	0.37 (0.16–0.87)	0.03	0.30 (0.13–0.70)	0.006	0.47 (0.21–1.08)	0.07
BRSC	0.33 (0.10–1.13)	0.08	0.42 (0.13–1.37)	0.15	1.02 (0.38–2.75)	0.97	1.25 (0.48–3.23)	0.64
CMS subtype^a^								
CMS1 vs. other cases	0.26 (0.04–1.93)	0.19	0.30 (0.04–2.21)	0.24	1.37 (0.41–4.59)	0.61	1.30 (0.38–4.51)	0.68
CMS2 vs. other cases	0.38 (0.12–1.25)	0.11	0.35 (0.11–1.14)	0.08	0.19 (0.03–1.40)	0.10	0.20 (0.03–1.46)	0.11
CMS3 vs. other cases	1.99 (1.00–3.99)	0.05	1.81 (0.85–3.87)	0.12	1.31 (0.52–3.30)	0.57	2.15 (0.72–6.45)	0.17
CMS4 vs. other cases	1.75 (0.90–3.39)	0.10	1.78 (0.89–3.55)	0.10	1.73 (0.77–3.89)	0.19	1.32 (0.55–3.13)	0.53
Unclassified vs. other cases	0.57 (0.22–1.48)	0.25	0.72 (0.28–1.88)	0.50	0.70 (0.24–2.04)	0.51	0.80 (0.27–2.39)	0.68

Abbreviation: N/A, not applicable.

a Adjusted analysis by pretreatment T stage, N stage, and cohort type (with Grampian as the reference group).

Molecular variables previously found to be associated with pCR at the FDR level were also analyzed in univariate and adjusted models by cohort and baseline T and N stages (Table 2). Only the RSS signature showed significant association for RFS in both univariate [OR = 0.35 (95% CI, 0.17–0.70; *P* = 0.003)] and adjusted models [OR = 0.37 (95% CI, 0.16–0.87; *P* = 0.03)]. RSS was also significantly associated with OS only in univariate analysis [OR = 0.30 (95% CI, 0.13–0.70; *P* = 0.006); Table 2]. A significant association was also found in T-TBRS for RFS in the univariate analysis [OR = 4.09 (95% CI, 1.11–15.02; *P* = 0.04)]. Overall, stromal signatures showed trends for poor survival. Finally, considering the interaction found between cytotoxic lymphocytes and CMS1/4 with the endpoint of pCR, we performed a similar analysis for RFS and OS that did not show any significant trend (Supplementary Table S6).

## Discussion

Response to RT treatments in rectal cancer is extremely variable, and no biomarker is being used clinically. Although gene expression profiling can aid the assessment of the tumor landscape to identify potential stratifiers, previous attempts to uncover transcriptomic signatures for RT response can be improved in particular in relation to statistical power and stratification by treatment (9).We recently published strong evidence that a new transcriptomic signature called RSS which may provide clinically relevant prediction of pCR to neoadjuvant RT combined with fluoropyrimidines in rectal cancer (9). In addition, analysis of preselected candidates found that pCR associated with cytotoxic lymphocytes, CMS1, and low F-TBRS, which were combined into the compound BRSC variable (9). In this follow-up study, these analyses have been expanded to consider different treatment regimens, explore additional relevant transcriptomic signatures, compare the prediction ability across specific CMS subtypes, and assess long-term survival. For this purpose, the largest transcriptomic dataset of rectal cancer pretreatment biopsies to date has been built and analyzed together with associated clinical data.

Although neoadjuvant RT is widely used to treat rectal cancer, this may be given in different specific regimens depending on the RT dose and additional cytotoxic chemotherapies such as Ox. To the best of our knowledge, the search for molecular predictors of RT has never used such relevant information, with only few studies curating patients to have one single RT regimen (9, 22). Here, we compare response rates in patients with locally advanced rectal cancer treated by RT+5FU with or without Ox and find high immune signals associated with pCR in both regimens. However, although enrichment in stromal signatures is associated with lack of pCR in 5FU-treated patients, a positive association of stromal signatures with treatment response was found in patients treated with Ox-containing regimens. Although patients in our amalgamated dataset were not randomized for treatment, most cases with these two regimens belong to the same two cohorts and show similar trends in immune markers. Although the effect level in patients treated with Ox may be small due to a low number of cases with pCR, the strong differential effect suggests that stromal activation is unlikely to be associated with lack of pCR upon addition of Ox. Evidence from the CAO/ARO/AIO-94 trial has demonstrated an improvement in the pCR rate and 3-year disease-free survival in the Ox chemoradiotherapy arm compared with the control chemoradiotherapy arm (25). Further research is needed to validate and understand our observation from a biological perspective. It is currently unclear which biological effects are caused by Ox treatment in the tumor microenvironment and even less about its combination with RT. Although our results may suggest defined biological effects of Ox or prolonged treatment, these may be explained by synergistic effects in combination with RT, which requires further investigation. Should these findings be validated, patients with high-stroma tumors may benefit from added Ox.

In our previous study, we found that the activation of TGFβ in fibroblasts associated with lack of pCR. Here, we added three other similar TGFβ signatures in endothelial cells, macrophages, and T cells for analysis (21). Overall, all four signatures showed similar outcome patterns. Although not surprising because all four correlate closely (21), this suggests that TGFβ activation in the microenvironment impacts response to treatment independent of the cellular source. Similar outcome patterns are also observed in analysis of several immune cell types, although not all of them: Monocytic and myeloid dendritic cell signatures do not show any signal in our curated Cap+RT subset. Interestingly, in this subset, an epithelial signature scoring ISCs was also associated with lack of pCR. Deep morphologic analyses looking at the presence and activation of microenvironment and tumor cells should be carried out to better understand the biology underlying these observations.

Given the link of tumor microenvironment signatures with pCR, we have performed a deeper exploratory analysis, showing that the associations of most immune and stromal markers with response to treatment are not strongly affected by the specific CMS subtype of the profiled tissue. Notably, an enrichment for the RSS signature showed strong predictive values for response in all CMS subtypes, suggesting that this biomarker may be useful in all patients without the need of further stratification, although it must be noted that a subset of the cohort was used to train RSS. However, an exception is the signature for cytotoxic lymphocytes that is associated with pCR, specifically in CMS1 and CMS4 subtypes and not in CMS2 or CMS3. These results are consistent with an independent role of CMS1, cytotoxic lymphocytes, and F-TBRS in RT response that we reported in a large subset of cases (9). They are also suggestive of a potential benefit of novel anticancer therapies targeting immune and stromal components in combination with chemoradiotherapy in this well-defined subset of patients. Given that CMS1 patients benefit from RT with fluoropyrimidines and CMS4 patients do not, further stratification may be considered based on the level of cytotoxic lymphocyte signatures. For example, patients with CMS4 biopsies who do not benefit from current SOC treatment may benefit from additional immunotherapy when levels of cytotoxic lymphocytes are high. However, no promising results were found for epithelial CMS2/CMS3 tumors, so much research is needed to identify new ways to tackle specifically these tumor subtypes not related with the microenvironment.

In order to better assess the benefit provided to patients, we have analyzed long-term survival in variables relevant in RT stratification in the neoadjuvant setting. Given the heterogeneity found in pCR depending on the additional chemotherapy, only cases treated with RT+5FU/Cap were analyzed. Reassuringly, pCR patients showed better RFS than non-pCR patients, providing support for the use of pathologic response as a surrogate endpoint. The only molecular variable showing improved survival was RSS, in accordance with the high frequency of pCR in cases with high RSS scores. Other variables showed signals in the expected direction, but the observed associations did not remain significant in adjusted models. Although this may be related to the modest statistical power in subgroup analyses with the low number of events, these results suggest that RSS could potentially perform better as a prognostic variable in colorectal cancer than other markers, including immune-related signatures.

Our study is limited by clinical heterogeneity resulting from the inclusion of opportunistic biopsies from patients with varying T and N stage statuses as well as various combinations of chemotherapeutic regimens, differences in radiation doses, fraction sizes and schedules, and varying timelines of conducting radical surgical excision after completion of neoadjuvant RT, which could be potential contributing factors affecting response outcomes. There were also limited numbers of patients within subgroups, which results in suboptimal statistical power for response analyses. Additionally, although Total Neoadjuvant Therapy (TNT) with chemotherapy may increasingly become an accepted SOC, our data predate the TNT era, and thus, examination of cases from TNT-specific clinical trials would be required to assess whether our predictors would be applicable to a TNT clinical population.

In summary, here we report early evidence that rectal cancers with high levels of stromal features may respond better to neoadjuvant RT upon addition of Ox. Furthermore, cytotoxic lymphocytes may show better response specifically in CMS1/CMS4 tumors. Future research in the clinical setting of neoadjuvant RT is likely to improve current lack of stratification. Finally, clinical data curation is essential to interrogate omic data and generate new avenues of research. Well-designed and statistically robust retrospective and prospective studies are essential for discovery science and provide useful insights on clinical relevance.

## Supplementary Material

Supplementary Figure 1 Supplementary Figure 1

Supplementary Figure 2 Supplementary Figure 2

Supplementary Figure 3 Supplementary Figure 3

Supplementary Figure 4 Supplementary Figure 4

Supplementary Figure 5 Supplementary Figure 5

Supplementary Table 1 Supplementary Table 1

Supplementary Table 2 Supplementary Table 2

Supplementary Table 3 Supplementary Table 3

Supplementary Table 4 Supplementary Table 4

Supplementary Table 5 Supplementary Table 5

Supplementary Table 6 Supplementary Table 6

Supplementary Material Supplementary Material

S:CORT members list S:CORT members list
