# Supplementary material for: Stratification to Neoadjuvant Radiotherapy in Rectal Cancer by Regimen and Transcriptional Signatures
Source: Cancer Res Commun. 2024 Jul 18;4(7):1765–76. doi: 10.1158/2767-9764.CRC-23-0502 (PMC11257085; doi:10.1158/2767-9764.CRC-23-0502)
Supplement: Supplementary Figure 1 [file crc-23-0502_supplementary_figure_1_suppsf1.docx]

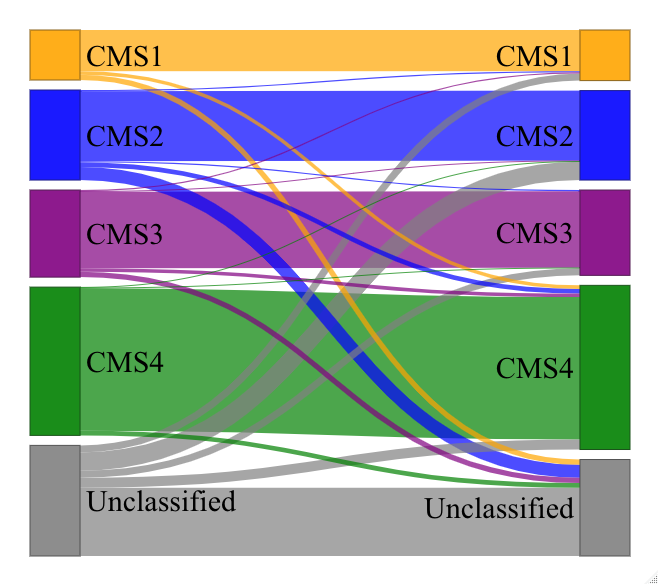


| **CMS subtypes in single cohort** | **CMS subtypes in combined dataset (Sveen et al.)** | | | | | **Total samples in single cohort** |
| --- | --- | --- | --- | --- | --- | --- |
|  | **CMS1** | **CMS2** | **CMS3** | **CMS4** | **Unclassified** |  |
| **CMS1** | 70 | 0 | 0 | 6 | 9 | **85** |
| **CMS2** | 2 | 119 | 2 | 8 | 22 | **153** |
| **CMS3** | 2 | 1 | 130 | 6 | 9 | **148** |
| **CMS4** | 0 | 1 | 1 | 242 | 8 | **252** |
| **Unclassified** | 12 | 31 | 12 | 17 | 116 | **188** |
| **Total samples in combined dataset** | **86** | **152** | **145** | **279** | **164** | **826** |

| **Single cohort**  **(Unclassified)** | **Combined cohort**  **(Unclassified)** | | McNemar’s X^2^ (1, N = 826) = 1.50,  *P* = 0.22 |
| --- | --- | --- | --- |
|  | No | Yes |  |
| No | 710 | 164 |  |
| Yes | 188 | 116 |  |

**Supplemental Figure 1A:** Sankey view comparing single vs combined cohorts for CMS subtype based on the CMScaller method.


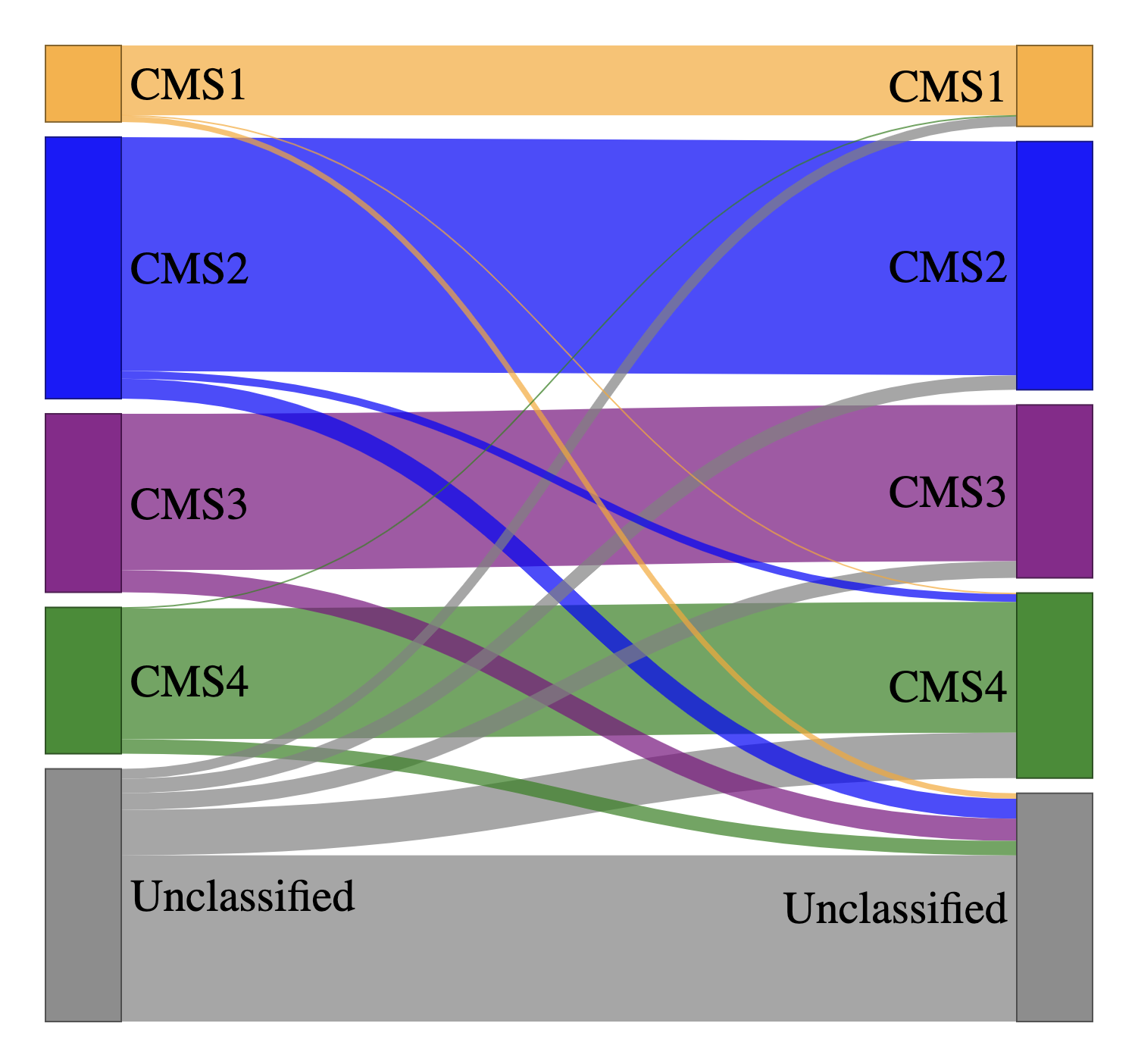


| **CMS subtypes in single cohort** | **CMS subtypes in combined dataset (Guinney et al.)** | | | | | **Total samples in single cohort** |
| --- | --- | --- | --- | --- | --- | --- |
|  | **CMS1** | **CMS2** | **CMS3** | **CMS4** | **Unclassified** |  |
| **CMS1** | 63 | 0 | 0 | 1 | 5 | **69** |
| **CMS2** | 0 | 211 | 0 | 7 | 18 | **236** |
| **CMS3** | 0 | 0 | 141 | 0 | 20 | **161** |
| **CMS4** | 1 | 0 | 0 | 118 | 13 | **132** |
| **Unclassified** | 9 | 13 | 15 | 41 | 150 | **228** |
| **Total samples in combined dataset** | **73** | **224** | **156** | **167** | **206** | **826** |

| **Single cohort**  **(Unclassified)** | **Combined cohort**  **(Unclassified)** | | McNemar’s X^2^ (1, N = 826) = 1.02,  *P* = 0.31 |
| --- | --- | --- | --- |
|  | No | Yes |  |
| No | 676 | 206 |  |
| Yes | 228 | 150 |  |

**Supplemental Figure 1B:** Sankey view comparing single vs combined cohorts for CMS subtype based on the CMSclassifier method.


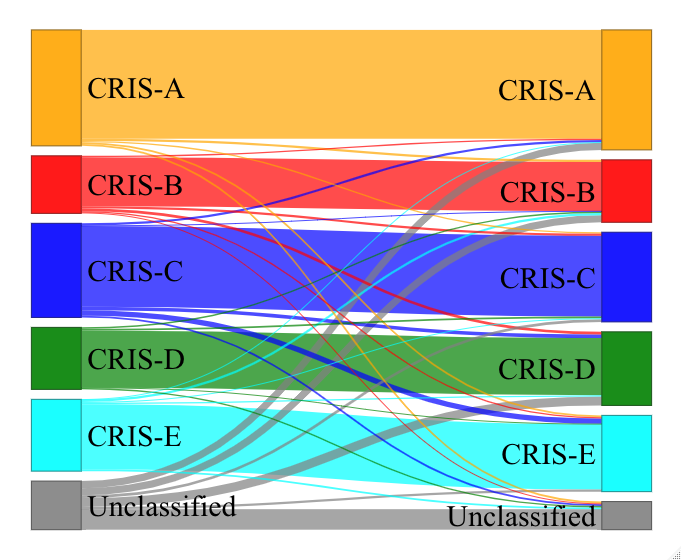


| **CRIS subtypes in single cohort** | **CRIS subtypes in combined dataset** | | | | | | **Total samples in single cohort** |
| --- | --- | --- | --- | --- | --- | --- | --- |
|  | **CRIS-A** | **CRIS-B** | **CRIS-C** | **CRIS-D** | **CRIS-E** | **Unclassified** |  |
| **CRIS-A** | 210 | 4 | 1 | 0 | 3 | 3 | **221** |
| **CRIS-B** | 2 | 93 | 2 | 5 | 3 | 1 | **106** |
| **CRIS-C** | 3 | 1 | 147 | 5 | 10 | 4 | **170** |
| **CRIS-D** | 0 | 3 | 3 | 108 | 1 | 2 | **117** |
| **CRIS-E** | 0 | 4 | 2 | 3 | 115 | 4 | **128** |
| **Unclassified** | 11 | 12 | 3 | 15 | 3 | 40 | **84** |
| **Total samples in combined dataset** | **226** | **117** | **158** | **136** | **135** | **54** | **826** |

| **Single cohort**  **(Unclassified)** | **Combined cohort**  **(Unclassified)** | | McNemar’s X^2^ (1, N = 826) = 6.0942,  *P* = 0.01 |
| --- | --- | --- | --- |
|  | No | Yes |  |
| No | 786 | 54 |  |
| Yes | 84 | 40 |  |

**Supplemental Figure 1C:** Sankey view comparing single vs combined cohorts for CRIS subtype.


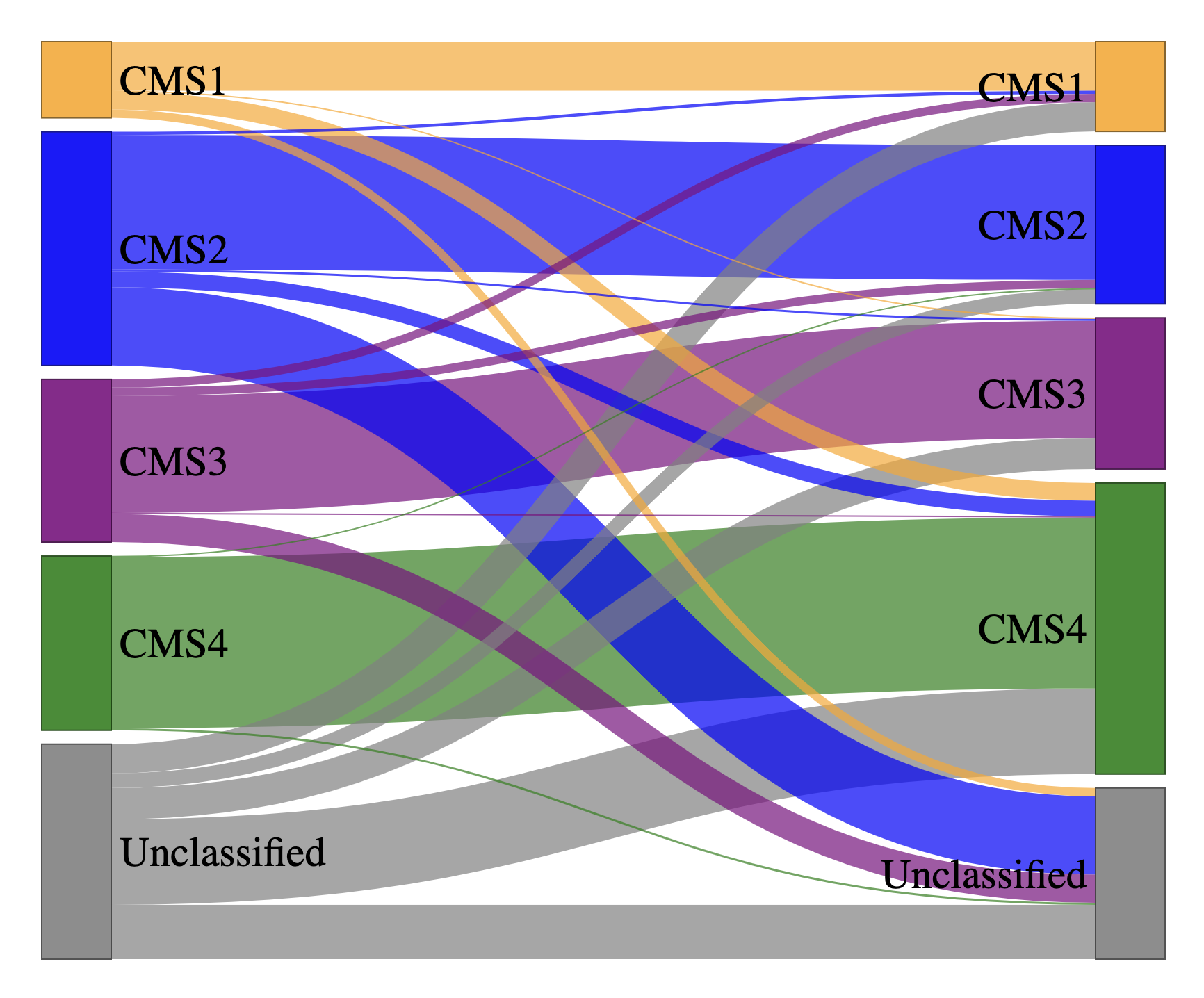


| **CMS subtypes in combined dataset (Guinney et al.)** | **CMS subtypes in combined dataset (Sveen et al.)** | | | | | **Total samples in combined dataset** |
| --- | --- | --- | --- | --- | --- | --- |
|  | **CMS1** | **CMS2** | **CMS3** | **CMS4** | **Unclassified** |  |
| **CMS1** | 47 | 0 | 1 | 17 | 8 | **73** |
| **CMS2** | 3 | 129 | 2 | 15 | 75 | **224** |
| **CMS3** | 8 | 8 | 112 | 1 | 27 | **156** |
| **CMS4** | 0 | 1 | 0 | 164 | 2 | **167** |
| **Unclassified** | 28 | 14 | 30 | 82 | 52 | **206** |
| **Total samples in combined dataset** | **86** | **152** | **145** | **279** | **164** | **826** |

**Supplemental Figure 1D:** Sankey view comparing combined cohorts for CMS subtypes (CMSclassifier vs CMScaller).


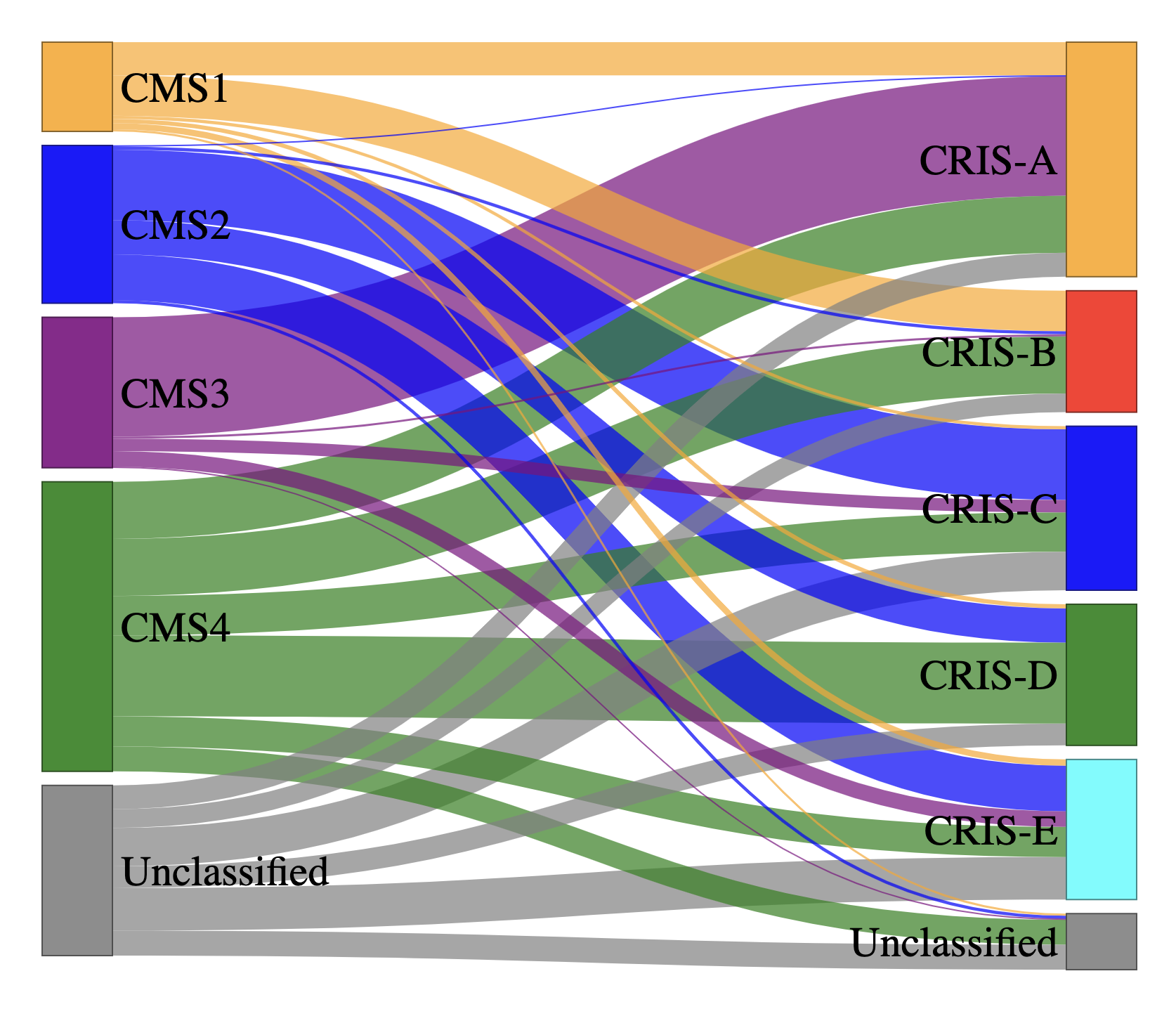


| **CMS subtypes in combined dataset** | **CRIS subtypes in combined dataset** | | | | | | **Total samples in combined dataset** |
| --- | --- | --- | --- | --- | --- | --- | --- |
|  | **CRIS-A** | **CRIS-B** | **CRIS-C** | **CRIS-D** | **CRIS-E** | **Unclassified** |  |
| **CMS1** | 32 | 39 | 3 | 4 | 6 | 2 | **86** |
| **CMS2** | 1 | 3 | 68 | 33 | 44 | 3 | **152** |
| **CMS3** | 115 | 2 | 12 | 0 | 15 | 1 | **145** |
| **CMS4** | 55 | 55 | 38 | 78 | 29 | 24 | **279** |
| **Unclassified** | 23 | 18 | 37 | 21 | 41 | 24 | **164** |
| **Total samples in combined dataset** | **226** | **117** | **158** | **136** | **135** | **54** | **826** |

**Supplemental Figure 1E:** Sankey view comparing combined cohorts for CMS (CMScaller) vs CRIS subtypes.
