# Supplementary material for: Stratification to Neoadjuvant Radiotherapy in Rectal Cancer by Regimen and Transcriptional Signatures
Source: Cancer Res Commun. 2024 Jul 18;4(7):1765–76. doi: 10.1158/2767-9764.CRC-23-0502 (PMC11257085; doi:10.1158/2767-9764.CRC-23-0502)
Supplement: Supplementary Figure 2 [file crc-23-0502_supplementary_figure_2_suppsf2.docx]

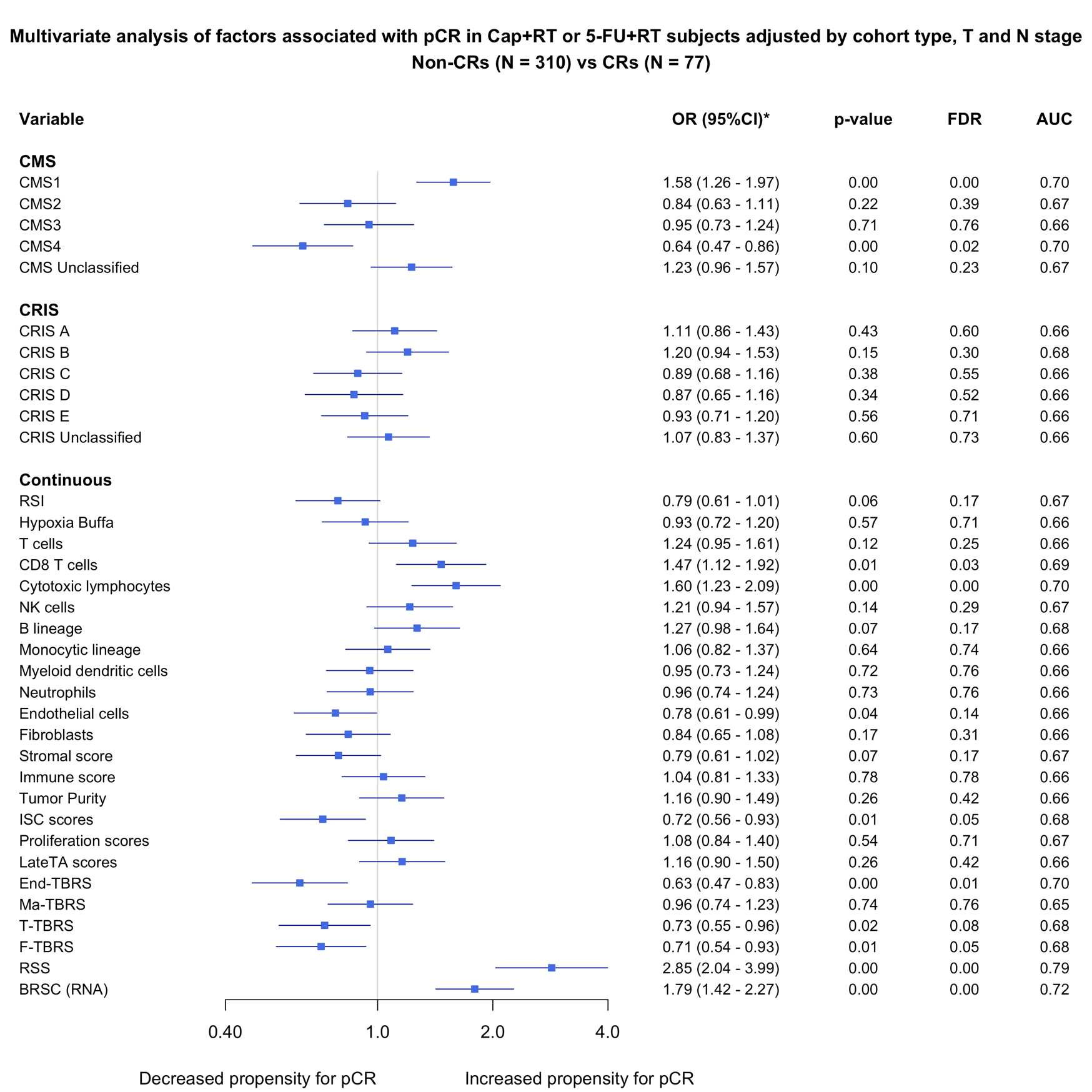


**Supplemental Figure 2A:** Multivariate logistic regression analysis of subjects receiving Cap+RT or 5-FU+RT depicted statistically significant associations of CMS1 and CMS4 subtypes, immune, stromal and radiosensitivity specific signatures with pCR.

* OR are reported as ‘OR per standard deviation’ to account for diverse distributions.

**
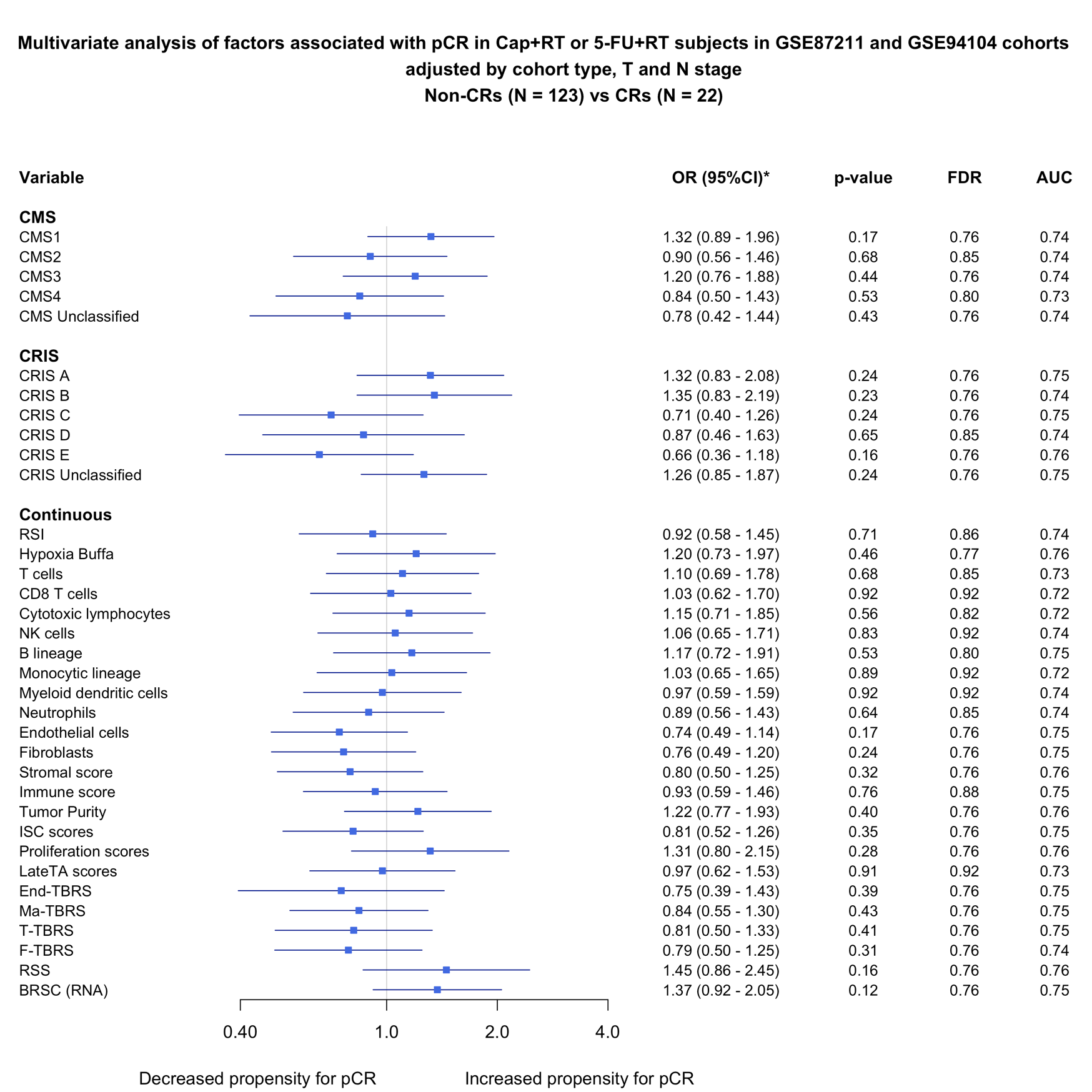
**

**Supplemental Figure 2B:** Multivariate logistic regression analysis of GSE87211 and GSE94104 cohorts receiving Cap+RT or 5-FU+RT did not demonstrate any significant association of clinical or transcriptomic signatures with pCR. However, the current analysis depicted a similar overall trend of association of signatures with pCR as noted in all cohorts receiving Cap+RT or 5-FU+RT.

* OR are reported as ‘OR per standard deviation’ to account for diverse distributions.
