# Supplementary material for: Stratification to Neoadjuvant Radiotherapy in Rectal Cancer by Regimen and Transcriptional Signatures
Source: Cancer Res Commun. 2024 Jul 18;4(7):1765–76. doi: 10.1158/2767-9764.CRC-23-0502 (PMC11257085; doi:10.1158/2767-9764.CRC-23-0502)
Supplement: Supplementary Figure 3 [file crc-23-0502_supplementary_figure_3_suppsf3.docx]

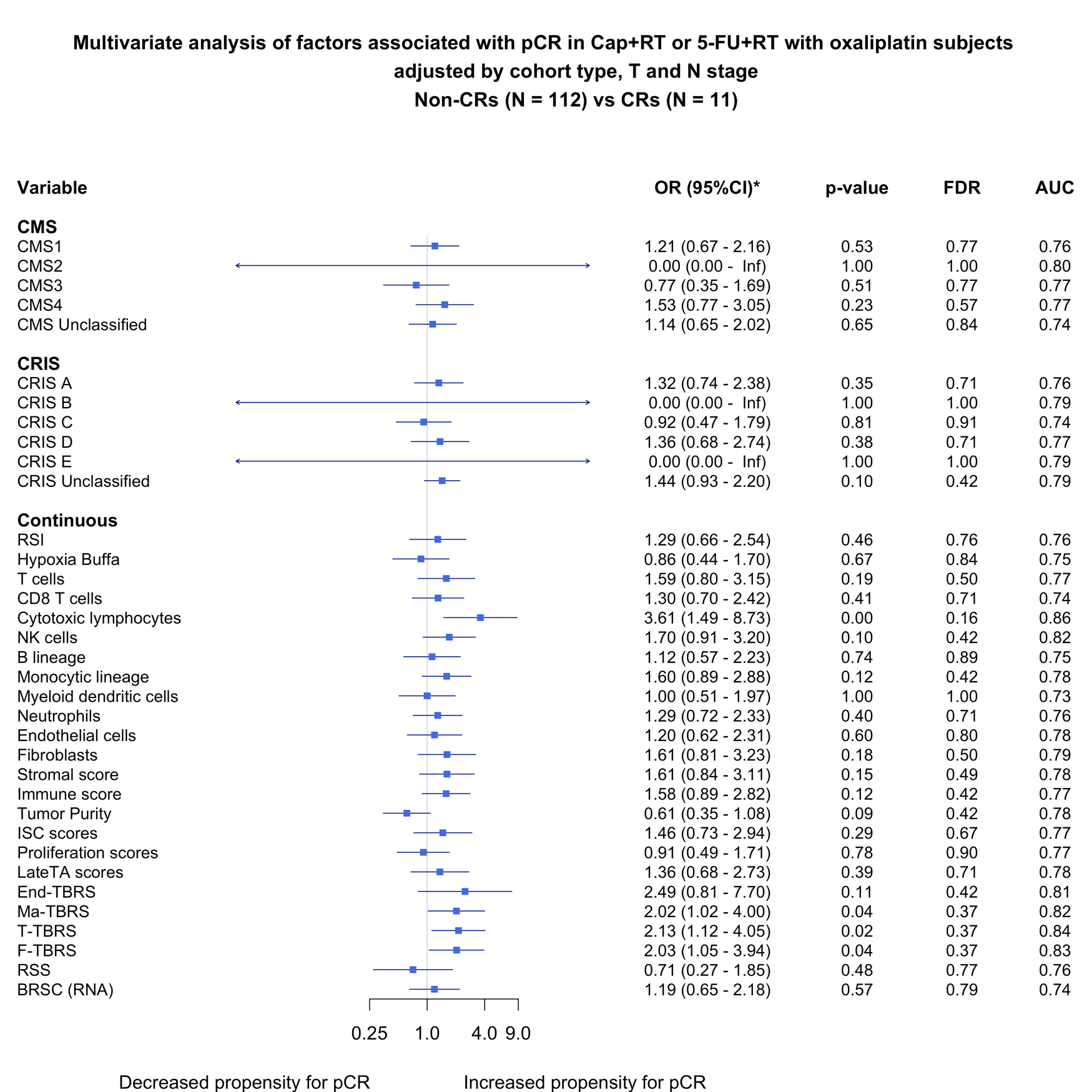


**Supplemental Figure 3:** Multivariate logistic regression analysis of subjects receiving Cap+RT or 5-FU+RT with oxaliplatin demonstrated statistically significant association of immune and stromal signatures with pCR.

* OR are reported as ‘OR per standard deviation’ to account for diverse distributions.
