# Supplementary material for: Stratification to Neoadjuvant Radiotherapy in Rectal Cancer by Regimen and Transcriptional Signatures
Source: Cancer Res Commun. 2024 Jul 18;4(7):1765–76. doi: 10.1158/2767-9764.CRC-23-0502 (PMC11257085; doi:10.1158/2767-9764.CRC-23-0502)
Supplement: Supplementary Figure 4 [file crc-23-0502_supplementary_figure_4_suppsf4.docx]

**
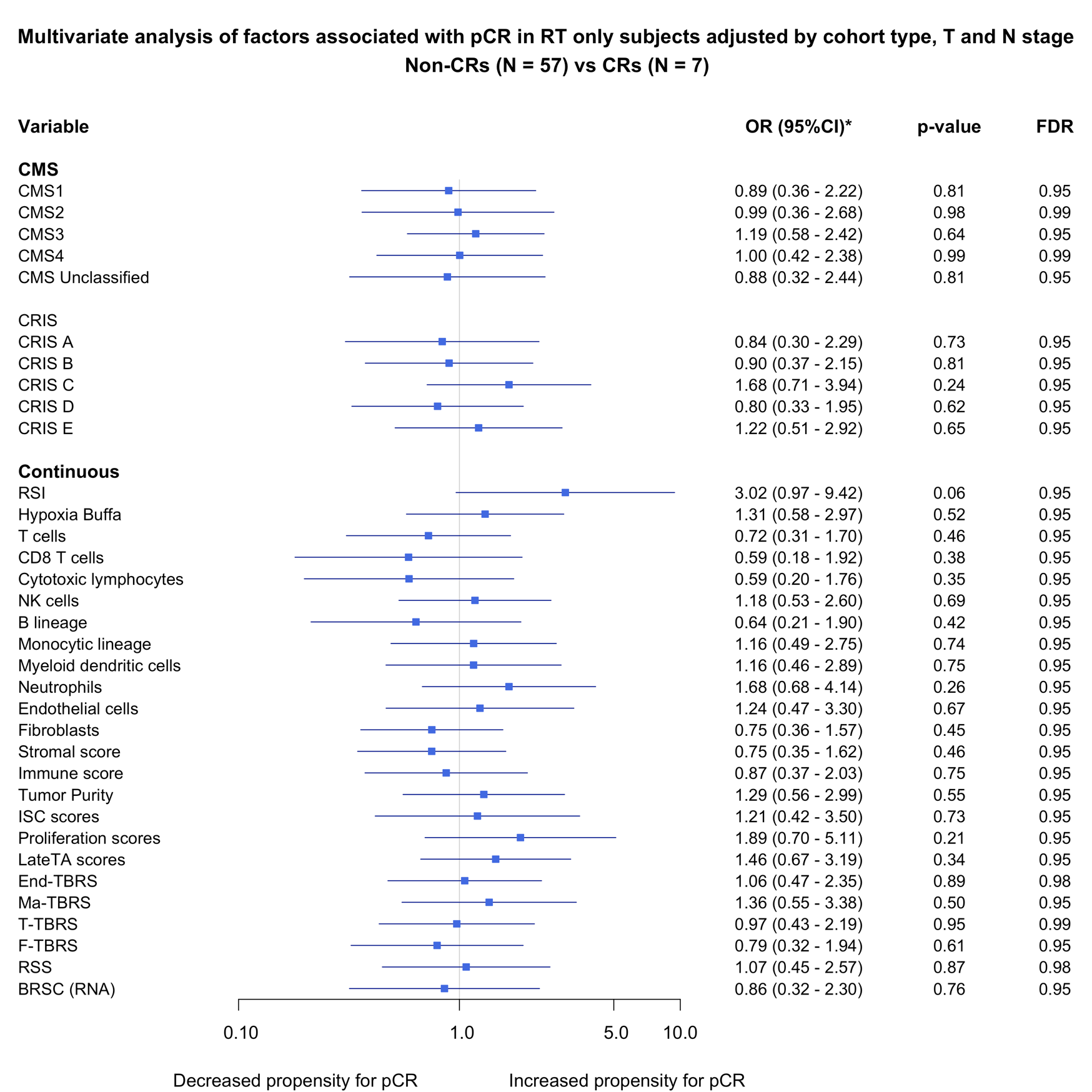
**

**Supplemental Figure 4:** Multivariate analysis of factors associated with pCR in RT only subjects did not demonstrate any significant association of clinical or transcriptomic signatures with pCR.

* OR are reported as ‘OR per standard deviation’ to account for diverse distributions. CRIS Unclassified was excluded
