# Supplementary material for: Stratification to Neoadjuvant Radiotherapy in Rectal Cancer by Regimen and Transcriptional Signatures
Source: Cancer Res Commun. 2024 Jul 18;4(7):1765–76. doi: 10.1158/2767-9764.CRC-23-0502 (PMC11257085; doi:10.1158/2767-9764.CRC-23-0502)
Supplement: Supplementary Figure 5 [file crc-23-0502_supplementary_figure_5_suppsf5.docx]

**
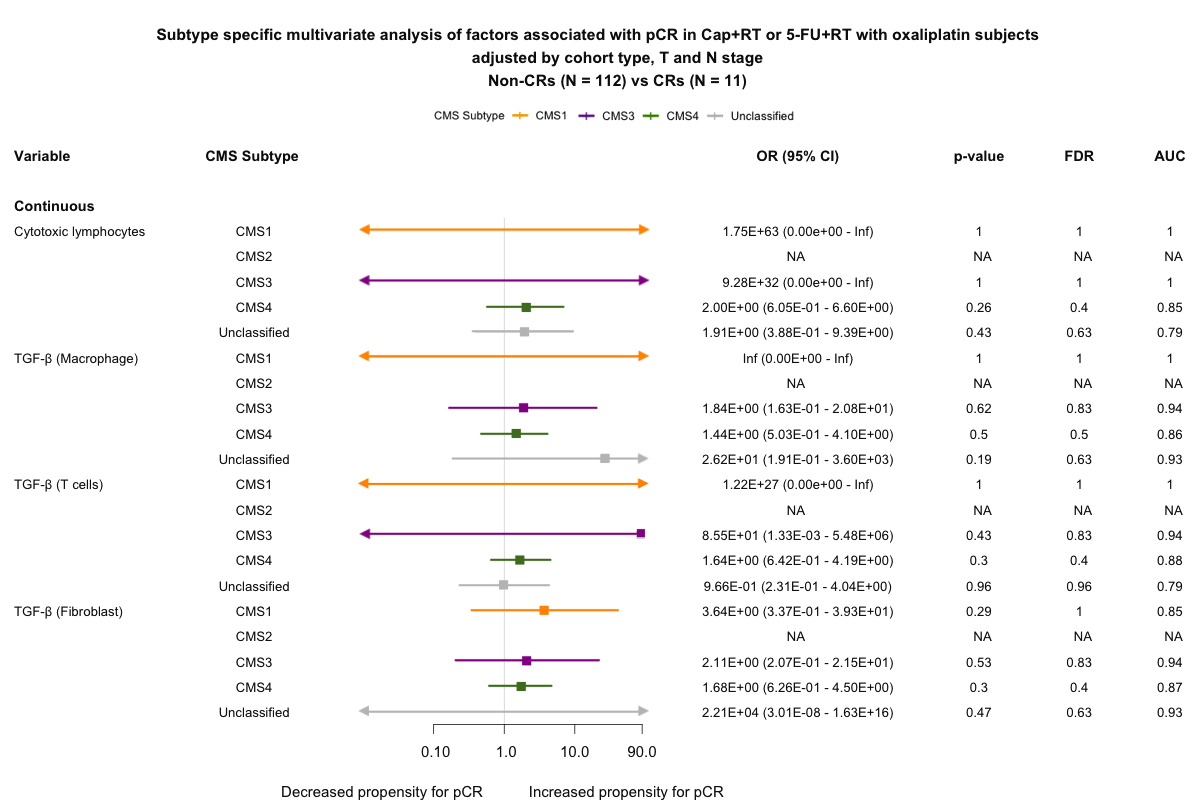
**

**Supplemental Figure 5:** Multivariate logistic regression results of selected variables from CMS based subtype specific analysis did not demonstrate any significant association of transcriptomic signatures with pCR in the RT-5FU/Cap + Ox cohort. Results corresponding to the CMS2 subtype are marked as NA since no patients in this treatment cohort were classified under this subtype.

* OR are reported as ‘OR per standard deviation’ to account for diverse distributions.
