# Supplementary material for: Stratification to Neoadjuvant Radiotherapy in Rectal Cancer by Regimen and Transcriptional Signatures
Source: Cancer Res Commun. 2024 Jul 18;4(7):1765–76. doi: 10.1158/2767-9764.CRC-23-0502 (PMC11257085; doi:10.1158/2767-9764.CRC-23-0502)
Supplement: Supplementary Table 1 [file crc-23-0502_supplementary_table_1_suppst1.docx]

**Supplemental Table S1:** Description of patient samples from all nine cohorts used in the current research study.

| **Cohort** | **Tumour biopsies** | **Tissue type** | **Platform** | **Genes (entrez id)** | **Neoadjuvant regimen(s)** |
| --- | --- | --- | --- | --- | --- |
| ARISTOTLE (Control Arm) | 121 | FFPE | Affymetrix Xcel | 24,426 | 45 Gy RT + capecitabine |
| COPERNICUS | 37 | FFPE | Affymetrix Xcel | 24,426 | Folfox then short-course RT |
| TREC | 37 | FFPE | Affymetrix Xcel | 24,426 | Short-course RT |
| GRAMPIAN | 223 | FFPE | Affymetrix Xcel | 24,426 | 45 Gy RT + capecitabine or 45Gy RT + capecitabine + oxaliplatin or 50Gy RT or 25Gy RT hypofractionated |
| GSE56699 | 57 | FFPE | Illumina HumanHT-12 WG-DASL V4.0 R2 | 20,310 | RT (no info on specific regimen) |
| GSE87211 | 203 | FF | Agilent-026652 Whole Human Genome Microarray 4x44K v2 | 21,755 | 45Gy RT + 5FU or 45Gy RT + 5FU + oxaliplatin or 45Gy RT + 5FU + oxaliplatin + cetuximab |
| GSE150082 | 39 | FF | Agilent-026652 Whole Human Genome Microarray 4x44K v2 | 21,755 | 45 Gy RT + capecitabine or capox then 45Gy RT + capecitabine |
| GSE94104 | 40 | FFPE | Illumina HumanHT-12 WG-DASL V4.0 R2 | 18,561 | 45 Gy RT + capecitabine or 45Gy RT + 5FU |
| GSE46862 | 69 | FF | Affymetrix Human Gene 1.0 ST Array | 18,830 | RT (no info on specific regimen) |
| **Combined Dataset** | **826** | **FFPE/FF** | **4 different platforms** | **15,985*** | - |

Abbreviations: FF, fresh frozen; FFPE, formalin-fixed paraffin-embedded. *Entrez gene ids in common across all platforms
