# Supplementary material for: Stratification to Neoadjuvant Radiotherapy in Rectal Cancer by Regimen and Transcriptional Signatures
Source: Cancer Res Commun. 2024 Jul 18;4(7):1765–76. doi: 10.1158/2767-9764.CRC-23-0502 (PMC11257085; doi:10.1158/2767-9764.CRC-23-0502)
Supplement: Supplementary Table 2 [file crc-23-0502_supplementary_table_2_suppst2.docx]

**Supplemental Table 2A:** Distribution of rectal cancer specimens by cohort type within the combined dataset based on pre-treatment clinical T and N stage status following neo-adjuvant radiation with or without chemotherapy.

|  | **Cohort Type** | | | | | | | | | |
| --- | --- | --- | --- | --- | --- | --- | --- | --- | --- | --- |
|  | ARISTOTLE (Control Arm) | COPERNICUS | TREC | Grampian | GSE56699 | GSE87211 | GSE94104 | GSE46862 | GSE150082 | **Total samples** |
| **T Stage** |  | | | | | | | | | |
| T1 | 0  (0.00%) | 0  (0.00%) | 7  (18.92%) | 1  (0.45%) | 0  (0.00%) | 0  (0.00%) | 0  (0.00%) | 0  (0.00%) | 0  (0.00%) | **8**  **(0.97%)** |
| T2 | 6  (4.96%) | 1  (2.70%) | 26  (70.27%) | 28  (12.56%) | 0  (0.00%) | 6  (2.96%) | 4  (10.00%) | 0  (0.00%) | 0  (0.00%) | **71**  **(8.60%)** |
| T3 | 94  (77.69%) | 35  (94.59%) | 0  (0.00%) | 152  (68.16%) | 0  (0.00%) | 184  (90.64%) | 18  (45.00%) | 0  (0.00%) | 0  (0.00%) | **483 (58.47%)** |
| T4 | 21  (17.36%) | 1  (2.70%) | 0  (0.00%) | 12  (5.38%) | 0  (0.00%) | 12  (5.91%) | 16  (40.00%) | 0  (0.00%) | 0  (0.00%) | **62**  **(7.51%)** |
| Missing | 0  (0.00%) | 0  (0.00%) | 4  (10.81%) | 30  (13.45%) | 57  (100.00%) | 1  (0.49%) | 2  (5.00%) | 69  (100.00%) | 39  (100.00%) | **202 (24.46%)** |
| **N Stage** |  | | | | | | | | | |
| N0 | 26  (21.49%) | 1  (2.70%) | 37  (100.00%) | 87  (39.01%) | 0  (0.00%) | 66  (32.51%) | 9  (22.50%) | 0  (0.00%) | 0  (0.00%) | **226 (27.36%)** |
| N1 | 57  (47.11%) | 26  (70.27%) | 0  (0.00%) | 82  (36.77%) | 0  (0.00%) | 133  (65.52%) | 15  (37.50%) | 0  (0.00%) | 0  (0.00%) | **313 (37.89%)** |
| N2 | 38  (31.40%) | 10  (27.03%) | 0  (0.00%) | 27  (12.11%) | 0  (0.00%) | 0  (0.00%) | 16  (40.00%) | 0  (0.00%) | 0  (0.00%) | **91**  **(11.02%)** |
| Missing | 0  (0.00%) | 0  (0.00%) | 0  (0.00%) | 27  (12.11%) | 57  (100.00%) | 4  (1.97%) | 0  (0.00%) | 69  (100.00%) | 39  (100.00%) | **196 (23.73%)** |
| **Total samples by cohort type** | 121  (14.65%) | 37  (4.48%) | 37  (4.48%) | 223  (27.00%) | 57  (6.90%) | 203  (24.58%) | 40  (4.84%) | 69  (8.35%) | 39  (4.72%) | **826 (100.00%)** |

**Supplemental Table 2B** Distribution of rectal cancer specimens by cohort type within the combined dataset based on pathological responses and treatment type following neo-adjuvant radiation with or without chemotherapy.

|  | **Cohort Type** | | | | | | | | | |
| --- | --- | --- | --- | --- | --- | --- | --- | --- | --- | --- |
|  | ARISTOTLE (Control Arm) | COPERNICUS | TREC | Grampian | GSE56699 | GSE87211 | GSE94104 | GSE46862 | GSE150082 | **Total samples** |
| **Pathological Response** |  |  |  |  |  |  |  |  |  |  |
| Complete Responders | 24  (19.83%) | 6  (16.22%) | 8  (21.62%) | 33  (14.8%) | 19  (33.33%) | 35  (17.24%) | 0  (0.00%) | 17  (24.64%) | 5  (12.82%) | **147 (17.80%)** |
| Non-Complete Responders | 97  (80.17%) | 31  (83.78%) | 24  (64.86%) | 190  (85.2%) | 37  (64.91%) | 168  (82.76%) | 40  (100.0%) | 52  (75.36%) | 28  (71.79%) | **667 (80.75%)** |
| Missing | 0  (0.00%) | 0  (0.00%) | 5  (13.51%) | 0  (0.00%) | 1  (1.75%) | 0  (0.00%) | 0  (0.00%) | 0  (0.00%) | 6  (15.38%) | **12**  **(1.45%)** |
| **Treatment Type** |  | | | | | | | | | |
| Capecitabine or Fluorouracil and Radiotherapy | 121  (100.00%) | 0  (0.00%) | 0  (0.00%) | 129  (57.85%) | 0  (0.00%) | 111 (54.68%) | 40 (100.00%) | 0  (0.00%) | 0  (0.00%) | **401 (48.55%)** |
| Capecitabine or Fluorouracil and Radiotherapy with Oxaliplatin | 0  (0.00%) | 0  (0.00%) | 0  (0.00%) | 50  (22.42%) | 0  (0.00%) | 87  (42.86%) | 0  (0.00%) | 0  (0.00%) | 0  (0.00%) | **137 (16.59%)** |
| Radiotherapy Alone | 0  (0.00%) | 0  (0.00%) | 37  (100.0%) | 44  (19.73%) | 0  (0.00%) | 0 (0.00%) | 0  (0.00%) | 0  (0.00%) | 0  (0.00%) | **81**  **(9.81%)** |
| Miscellaneous Chemoradiation | 0  (0.00%) | 37  (100.00%) | 0  (0.00%) | 0  (0.00%) | 57 (100.0%) | 5  (2.46%) | 0  (0.00%) | 69  (100.0%) | 39  (100.00%) | **207 (25.06%)** |
