# Supplementary material for: Stratification to Neoadjuvant Radiotherapy in Rectal Cancer by Regimen and Transcriptional Signatures
Source: Cancer Res Commun. 2024 Jul 18;4(7):1765–76. doi: 10.1158/2767-9764.CRC-23-0502 (PMC11257085; doi:10.1158/2767-9764.CRC-23-0502)
Supplement: Supplementary Table 3 [file crc-23-0502_supplementary_table_3_suppst3.docx]

**Supplemental Table 3:** Distribution of rectal cancer specimens by cohort type within the combined dataset based on CMS and CRIS subtypes following neo-adjuvant radiation with or without chemotherapy.

|  | **Cohort Type** | | | | | | | | | |
| --- | --- | --- | --- | --- | --- | --- | --- | --- | --- | --- |
|  | ARISTOTLE (Control Arm) | COPERNICUS | TREC | Grampian | GSE56699 | GSE87211 | GSE94104 | GSE46862 | GSE150082 | **Total samples** |
| **CMS Subtype** | X^2^ (24, N = 662) = 34.082, *P* = 0.08 (excluding Unclassified samples) | | | | | | | | | |
| CMS1 | 17  (14.05%) | 4  (10.81%) | 5  (13.51%) | 27  (12.11%) | 3  (5.26%) | 18  (8.87%) | 2  (5.00%) | 5  (7.25%) | 5  (12.82%) | **86**  **(10.41%)** |
| CMS2 | 17  (14.05%) | 10  (27.03%) | 7  (18.92%) | 38  (17.04%) | 15  (26.32%) | 37  (18.23%) | 3  (7.50%) | 17  (24.64%) | 8  (20.51%) | **152**  **(18.40%)** |
| CMS3 | 18  (14.88%) | 3  (8.11%) | 8  (21.62%) | 28  (12.56%) | 13  (22.81%) | 53  (26.11%) | 9  (22.50%) | 9  (13.04%) | 4  (10.26%) | **145 (17.55%)** |
| CMS4 | 43  (35.54%) | 11  (29.73%) | 11  (29.73%) | 79  (35.43%) | 15  (26.32%) | 66  (32.51%) | 15  (37.50%) | 24  (34.78%) | 15  (38.46%) | **279**  **(33.78%)** |
| Unclassified | 26  (21.49%) | 9  (24.32%) | 6  (16.22%) | 51  (22.87%) | 11  (19.30%) | 29  (14.29%) | 11  (27.50%) | 14  (20.29%) | 7  (17.95%) | **164 (19.85%)** |
| **CRIS Subtype** | X^2^ (32, N = 772) = 35.188, *P* = 0.32 (excluding Unclassified samples) | | | | | | | | | |
| CRIS-A | 28  (23.14%) | 5  (13.51%) | 7  (18.92%) | 52  (23.32%) | 17  (29.82%) | 63 (31.03%) | 12  (30.00%) | 27  (39.13%) | 15  (38.46%) | **226 (27.36%)** |
| CRIS-B | 20  (16.53%) | 7  (18.92%) | 5  (13.51%) | 33  (14.80%) | 8  (14.04%) | 25  (12.32%) | 8  (20.00%) | 6  (8.70%) | 5  (12.82%) | **117 (14.16%)** |
| CRIS-C | 27  (22.31%) | 11  (29.73%) | 10  (27.03%) | 47  (21.08%) | 9  (15.79%) | 28  (13.79%) | 5  (12.50%) | 15  (21.74%) | 6  (15.38%) | **158 (19.13%)** |
| CRIS-D | 17  (14.05%) | 6  (16.22%) | 7  (18.92%) | 45  (20.18%) | 10  (17.54%) | 31  (15.27%) | 9  (22.50%) | 6  (8.70%) | 5  (12.82%) | **136 (16.46%)** |
| CRIS-E | 19  (15.70%) | 8  (21.62%) | 6  (16.22%) | 36  (16.14%) | 8  (14.04%) | 39  (19.21%) | 3  (7.50%) | 10  (14.49%) | 6  (15.38%) | **135 (16.34%)** |
| Unclassified | 10  (8.26%) | 0  (00.00%) | 2  (5.41%) | 10  (4.48%) | 5  (8.77%) | 17  (8.37%) | 3  (7.50%) | 5  (7.25%) | 2  (5.13%) | **54**  **(6.54%)** |
