# Supplementary material for: Stratification to Neoadjuvant Radiotherapy in Rectal Cancer by Regimen and Transcriptional Signatures
Source: Cancer Res Commun. 2024 Jul 18;4(7):1765–76. doi: 10.1158/2767-9764.CRC-23-0502 (PMC11257085; doi:10.1158/2767-9764.CRC-23-0502)
Supplement: Supplementary Table 4 [file crc-23-0502_supplementary_table_4_suppst4.docx]

**Supplemental Table 4A**: Distribution of rectal cancer specimens by CMS subtype within the combined dataset based on pre-treatment clinical T stage status. X^2^ (9, N = 500) = 11.609, *P* = 0.24 (excluding missing and Unclassified samples).

| **T Stage** | **CMS Subtype** | | | | | **Total** |
| --- | --- | --- | --- | --- | --- | --- |
|  | CMS1 | CMS2 | CMS3 | CMS4 | Unclassified |  |
| T1 | 1 (1.16%) | 4 (2.63%) | 1 (0.69%) | 1 (0.36%) | 1 (0.61%) | 8 |
| T2 | 9 (10.47%) | 8 (5.26%) | 18 (12.41%) | 22 (7.89%) | 14 (8.54%) | 71 |
| T3 | 54 (62.79%) | 82 (53.95%) | 83 (57.24%) | 163 (58.42%) | 101 (61.59%) | 483 |
| T4 | 6 (6.98%) | 12 (7.89%) | 9 (6.21%) | 27 (9.68%) | 8 (4.88%) | 62 |
| Missing | 16 (18.60%) | 46 (30.26%) | 34 (23.45%) | 66 (23.66%) | 40 (24.39%) | 202 |
| **Total samples**  **by subtype** | **86 (100.00%)** | **152 (100.00%)** | **145 (100.00%)** | **279 (100.00%)** | **164 (100.00%)** |  |

**Supplemental Table 4B**: Distribution of rectal cancer specimens by CRIS subtype within the combined dataset based on pre-treatment clinical T stage status. X^2^ (12, N = 584) = 16.28, *P* = 0.18 (excluding missing and unclassified samples).

| **T Stage** | **CRIS Subtype** | | | | | | **Total** |
| --- | --- | --- | --- | --- | --- | --- | --- |
|  | CRIS-A | CRIS-B | CRIS-C | CRIS-D | CRIS-E | Unclassified |  |
| T1 | 1 (0.44%) | 1 (0.85%) | 3 (1.90%) | 2 (1.47%) | 1 (0.74%) | 0 (0.00%) | 8 |
| T2 | 21 (9.29%) | 8 (6.84%) | 17 (10.76%) | 14 (10.29%) | 8 (5.93%) | 3 (5.56%) | 71 |
| T3 | 118 (52.21%) | 76 (64.96%) | 93 (58.86%) | 75 (55.15%) | 91 (67.41%) | 30 (55.56%) | 483 |
| T4 | 15 (6.64%) | 8 (6.84%) | 7 (4.43%) | 18 (13.24%) | 7 (5.19%) | 7 (12.96%) | 62 |
| Missing | 71 (31.42%) | 24 (20.51%) | 38 (24.05%) | 27 (19.85%) | 28 (20.74%) | 14 (25.93%) | 202 |
| **Total samples by subtype** | **226 (100.00%)** | **117 (100.00%)** | **158 (100.00%)** | **136 (100.00%)** | **135 (100.00%)** | **54 (100.00%)** |  |
