# Supplementary material for: Stratification to Neoadjuvant Radiotherapy in Rectal Cancer by Regimen and Transcriptional Signatures
Source: Cancer Res Commun. 2024 Jul 18;4(7):1765–76. doi: 10.1158/2767-9764.CRC-23-0502 (PMC11257085; doi:10.1158/2767-9764.CRC-23-0502)
Supplement: Supplementary Table 5 [file crc-23-0502_supplementary_table_5_suppst5.docx]

**Supplemental Table 5A**: Distribution of rectal cancer specimens by CMS subtype within the combined dataset based on pre-treatment clinical N stage status. X^2^ (6, N = 503) = 20.257, *P* = 0.003 (excluding missing and unclassified samples).

| **N Stage** | **CMS Subtype** | | | | | **Total** |
| --- | --- | --- | --- | --- | --- | --- |
|  | CMS1 | CMS2 | CMS3 | CMS4 | Unclassified |  |
| N0 | 31 (36.05%) | 32 (21.05%) | 52 (35.86%) | 61 (21.86%) | 50 (30.49%) | 226 |
| N1 | 26 (30.23%) | 61 (40.13%) | 53 (36.55%) | 115 (41.22%) | 58 (35.37%) | 313 |
| N2 | 13 (15.12%) | 13 (8.55%) | 8 (5.52%) | 38 (13.62%) | 19 (11.59%) | 91 |
| Missing | 16 (18.60%) | 46 (30.26%) | 32 (22.07%) | 65 (23.30%) | 37 (22.56%) | 196 |
| **Total samples by subtype** | **86 (100.00%)** | **152 (100.00%)** | **145 (100.00%)** | **279 (100.00%)** | **164 (100.00%)** |  |

**Supplemental Table 5B**: Distribution of rectal cancer specimens by CRIS subtype within the combined dataset based on pre-treatment clinical N stage status. X^2^ (8, N = 588) = 28.763, *P* < 0.001 (excluding missing and unclassified samples).

| **N Stage** | **CRIS Subtype** | | | | | | **Total** |
| --- | --- | --- | --- | --- | --- | --- | --- |
|  | CRIS-A | CRIS-B | CRIS-C | CRIS-D | CRIS-E | Unclassified |  |
| N0 | 67 (29.65%) | 30 (25.64%) | 48 (30.38%) | 25 (18.38%) | 37 (27.41%) | 19 (35.19%) | 226 |
| N1 | 73 (32.30%) | 37 (31.62%) | 58 (36.71%) | 61 (44.85%) | 63 (46.67%) | 21 (38.89%) | 313 |
| N2 | 17 (7.52%) | 24 (20.51%) | 17 (10.76%) | 23 (16.91%) | 8 (5.93%) | 2 (3.70%) | 91 |
| Missing | 69 (30.53%) | 26 (22.22%) | 35 (22.15%) | 27 (19.85%) | 27 (20.00%) | 12 (22.22%) | 196 |
| **Total samples by subtype** | **226 (100.00%)** | **117 (100.00%)** | **158 (100.00%)** | **136 (100.00%)** | **135 (100.00%)** | **54 (100.00%)** |  |
