# Supplementary material for: Stratification to Neoadjuvant Radiotherapy in Rectal Cancer by Regimen and Transcriptional Signatures
Source: Cancer Res Commun. 2024 Jul 18;4(7):1765–76. doi: 10.1158/2767-9764.CRC-23-0502 (PMC11257085; doi:10.1158/2767-9764.CRC-23-0502)
Supplement: Supplementary Table 6 [file crc-23-0502_supplementary_table_6_suppst6.docx]

**Supplemental Table 6:** CMS subtype specific RFS and OS outcomes in combined Grampian and GSE87211 cohorts (Cap+RT/5-FU+RT subjects).

|  | **RFS** | | | | **OS** | | | |
| --- | --- | --- | --- | --- | --- | --- | --- | --- |
|  | **Univariate HR (95% CI)** | **p-value** | **Multivariate HR (95% CI)*** | **p-value** | **Univariate HR (95% CI)** | **p-value** | **Multivariate HR (95% CI)*** | **p-value** |
| Cytotoxic Lymphocytes (CMS1 and CMS4 vs other cases) | 0.45 (0.07 - 2.81) | 0.39 | 0.40 (0.06 - 2.87) | 0.36 | 0.38 (0.04 - 3.54) | 0.40 | 0.39 (0.03 - 4.55) | 0.45 |

* Multivariate analysis was adjusted by T stage, N stage, and Cohort Type (with Grampian as the reference group).
