# Supplementary material for: Stratification to Neoadjuvant Radiotherapy in Rectal Cancer by Regimen and Transcriptional Signatures
Source: Cancer Res Commun. 2024 Jul 18;4(7):1765–76. doi: 10.1158/2767-9764.CRC-23-0502 (PMC11257085; doi:10.1158/2767-9764.CRC-23-0502)
Supplement: Supplementary Material [file crc-23-0502_supplementary_material_suppsm.docx]

**Supplementary Material and Methods (Mahmood et al)**

**Clinical cohorts**

ARISTOTLE is a clinical Phase 3 trial (EudraCT number 2008-005782-59) of advanced rectal cancer with standard neoadjuvant therapy (radiotherapy and capecitabine) or with addition of irinotecan followed by total mesorectal excision. Patients previously receiving radiotherapy or presenting metastatic disease were excluded. Out of the 580 patients randomised, 121 patients from the control arm of ARISTOTLE were eligible and available for analysis, who received capecitabine 5 days a week for 5 weeks in addition to radiotherapy of 45 Gray in 25 fractions over the course of 5 weeks. Unlocked clinical data was provided by the ARISTOTLE study team.

COPERNICUS was a Phase 2 study (EudraCT number 2010-023083-40 ) evaluating the use of sequential chemotherapy and then radiation followed by immediate curative surgery for operable rectal cancer (1). Patients underwent four cycles of chemotherapy; and received oxaliplatin 85mg/m² IV plus levofolinic acid (or equivalent) 175 mg (concurrent, over 2 hours), then fluorouracil (5-FU) 400 mg/m² (bolus), then fluorouracil 2400 mg/m² (continuous IV infusion over 46 hours) on a 2-weekly cycle. Within a two-week period, patients began short course pre-operative radiotherapy (SCPRT) treatment prescribed at 25 Gray in 5 fractions over 5-7 days. Surgical resection and pathological response evaluation was performed within a week after completing radiotherapy. 37 out of 60 registered patients were available for analysis.

TREC is a Phase 2 clinical trial (ISRCTN number: 14422743) which compared transanal endoscopic microsurgery (TEM) with SCPRT, where the latter was followed by delayed local excision with TEM (after an 8 – 10 week interval) for patients with early (T1 or T2 N0) rectal cancer (2). Patients on the SCPRT arm received a total of 25 Gy in 5 fractions over the course of 5 days. 37 out of 55 randomised patients were available for analysis.

The Grampian dataset comprised patients treated in Aberdeen Royal Infirmary with MRI defined LARC receiving SOC neoadjuvant radiotherapy with or without chemotherapy. Most subjects (N=129) received capecitabine alone with pelvic radiotherapy of 45 Gy in 25 fractions over 5 weeks. Remaining individuals (n=94) received the combination of capecitabine (500, 650, 825mg/m^2^ twice daily, 7 days a week during radiation), oxaliplatin (130mg/m^2^, days 1 and 29) and radiotherapy (45 Gy over 25 days) or radiotherapy alone in either 50 Gy in 25 fractions or a hypofractionated course of 25 Gy in 5 fractions.

Regarding public repositories, GSE56699 consisted of rectal cancer patients who received preoperative radiotherapy (3). Subjects in GSE87211 cohort received neoadjuvant chemoradiotherapy comprising either 5-FU, 5-FU with oxaliplatin or 5-FU with oxaliplatin and cetuximab as chemotherapeutic agents (4). Patients from GSE150082 with LARC received standard long course radiotherapy (LCRT) consisting of 50.4 Gy in 28 fractions (delivered as 1.8 Gy per fraction) with concurrent capecitabine (825 mg/m^2^/bid for 28 days) (5). Subjects presenting with high risk features for systemic relapse such as extramural vascular invasion, high mesorectal node burden and lateral lymph node dissection underwent total neo-adjuvant therapy (TNT) which included three cycles of capecitabine with oxaliplatin (130 mg/m² of oxaliplatin on day 1 and capecitabine 1000 mg/m²/bid, days 1-14 every 3 weeks) before standard LCRT with concurrent capecitabine. All patients then received two additional cycles of capecitabine (850 mg/m²/bid, days 1-14 every 3 weeks) until response assessment was performed. Similarly, LARC patients on the GSE94104 cohort also received LCRT comprising 45 Gy in 25 fractions over 5 weeks with capecitabine or 5-fluorouracil (6). Finally, patients in GSE46862 cohort received pre-operative chemoradiation for their LARC, although treatment regimens were not available.

**References**

1. Gollins S, West N, Sebag-Montefiore D, Susnerwala S, Falk S, Brown N, et al. A prospective phase II study of pre-operative chemotherapy then short-course radiotherapy for high risk rectal cancer: COPERNICUS. Br J Cancer. 2018;119:697–706.

2. Bach SP, Gilbert A, Brock K, Korsgen S, Geh I, Hill J, et al. Radical surgery versus organ preservation via short-course radiotherapy followed by transanal endoscopic microsurgery for early-stage rectal cancer (TREC): a randomised, open-label feasibility study. Lancet Gastroenterol Hepatol. 2020;6:92–105.

3. Isella C, Terrasi A, Bellomo SE, Petti C, Galatola G, Muratore A, et al. Stromal contribution to the colorectal cancer transcriptome. Nature Genetics. 2015;47:312–9.

4. Hu Y, Gaedcke J, Emons G, Beissbarth T, Grade M, Jo P, et al. Colorectal cancer susceptibility loci as predictive markers of rectal cancer prognosis after surgery. Genes Chromosomes and Cancer. 2018;57:140–9.

5. Sendoya JM, Iseas S, Coraglio M, Golubicki M, Robbio J, Salanova R, et al. Pre-Existing Tumoral B Cell Infiltration and Impaired Genome Maintenance Correlate with Response to Chemoradiotherapy in Locally Advanced Rectal Cancer. Cancers (Basel). 2020;12:2227.

6. Alderdice M, Richman SD, Gollins S, Stewart JP, Hurt C, Adams R, et al. Prospective patient stratification into robust cancer-cell intrinsic subtypes from colorectal cancer biopsies. Journal of Pathology. 2018;245:19–28.
