# Supplementary material for: Stratification to Neoadjuvant Radiotherapy in Rectal Cancer by Regimen and Transcriptional Signatures
Source: Cancer Res Commun. 2024 Jul 18;4(7):1765–76. doi: 10.1158/2767-9764.CRC-23-0502 (PMC11257085; doi:10.1158/2767-9764.CRC-23-0502)
Supplement: S:CORT members list [file crc-23-0502_scort_members_list_suppsm1.docx]

**S:CORT consortium author list:**

Andrew Blake^1^,

Francesca M Buffa^1,2^,

Enric Domingo ^1,3^,

Geoffrey Higgins^1^,

Christopher Holmes^1^,

Viktor H Koelzer^1,4^,

Simon J Leedham^1^,

Timothy S Maughan^1,5^,

Gillies McKenna^1^,

James Robineau^1^,

Ian Tomlinson^1^,

Michael Youdell^1^,

Philip Quirke^6^,

Susan D Richman^6^,

David Sebag-Montefiore^6^,

Matthew Seymour^6^,

Nicholas P West^6^,

Philip D Dunne^7^,

Richard Kennedy^7^,

Mark Lawler^7^,

Keara L Redmond^7^,

Manuel Salto-Tellez^7^,

Peter Campbell^8^,

Aikaterini Chatzipli^8^,

Claire Hardy^8^,

Ultan McDermott^8^,

Simon P Bach^9^,

Andrew Beggs^9^,

Jean-Baptiste Cazier^9^,

Gary Middleton^9^,

Dion Morton^9^,

Celina Whalley^9^,

Louise Brown^10^,

Richard Kaplan^10^,

Graeme Murray^11^,

Richard Wilson^12^,

Richard Adams^13^,

Richard Sullivan^14^,

Leslie Samuel^15^,

Paul Harkin^16^,

Steven Walker^16^,

Jim Hill Christie^17^,

Chieh-Hsi Wu^18^,

Dennis Horgan ^19^,

^1^University of Oxford, Oxford, United Kingdom

^2^Bocconi University, Milan, Italy

^3^Cancer Research UK Scotland Centre, Glasgow and Edinburgh, United Kingdom

^4^University of Bern, Bern, Switzerland

^5^University of Liverpool, Liverpool, United Kingdom

^6^University of Leeds, Leeds, United Kingdom

^7^Queen’s University Belfast, Belfast, United Kingdom

^8^Wellcome Trust Sanger Institute, Hinxton, Cambridge, United Kingdom

^9^University of Birmingham, Birmingham, United Kingdom

^10^University College London, London, United Kingdom

^11^University of Aberdeen, Aberdeen, United Kingdom

^12^University of Glasgow, Glasgow, United Kingdom

^13^University of Cardiff, Glasgow, United Kingdom

^14^Kings College London, London, United Kingdom

^15^Grampian NHS Health Board, United Kingdom

^16^Almac group, Craigavon, United Kingdom

^17^Hospital Manchester, Manchester, United Kingdom

^18^Southampton University, Southampton, United Kingdom

^19^European Association of Precision Medicine
